# The Global Prevalence of Daptomycin, Tigecycline, and Linezolid-Resistant *Enterococcus faecalis* and *Enterococcus faecium* Strains From Human Clinical Samples: A Systematic Review and Meta-Analysis

**DOI:** 10.3389/fmed.2021.720647

**Published:** 2021-09-10

**Authors:** Masoud Dadashi, Parastoo Sharifian, Nazila Bostanshirin, Bahareh Hajikhani, Narjess Bostanghadiri, Nafiseh Khosravi-Dehaghi, Alex van Belkum, Davood Darban-Sarokhalil

**Affiliations:** ^1^Department of Microbiology, School of Medicine, Alborz University of Medical Sciences, Karaj, Iran; ^2^Non-communicable Diseases Research Center, Alborz University of Medical Sciences, Karaj, Iran; ^3^Department of Microbiology, School of Medicine, Shahid Beheshti University of Medical Sciences, Tehran, Iran; ^4^Department of Microbiology, School of Medicine, Iran University of Medical Sciences, Tehran, Iran; ^5^Department of Pharmacognosy, School of Pharmacy, Alborz University of Medical Sciences, Karaj, Iran; ^6^Evidence-Based Phytotherapy and Complementary Medicine Research Center, Alborz University of Medical Sciences, Karaj, Iran; ^7^Data Analytics Unit, bioMérieux, La Balme-les-Grottes, France

**Keywords:** *E. faecalis*, *E. faecium*, tigecyclin, daptomycin, linezolid, resistant, meta-analysis

## Abstract

**Background and Aim:** The predominant species of the *Enterococcus, Enterococcus faecalis* (*E. faecalis*) and *Enterococcus faecium* (*E. faecium*) cause great variety of infections. Therefore, the expansion of antimicrobial resistance in the *Enterococcus* is one of the most important global concerns. This study was conducted to investigate the prevalence of resistance to linezolid, tigecycline, and daptomycin among enterococcal strains isolated from human clinical specimens worldwide.

**Methods:** Several databases including Web of Science, EMBASE, and Medline (*via* PubMed), were carefully searched and reviewed for original research articles available in databases and published between 2000 and 2020. A total of 114 studies worldwide that address *E. faecalis* and *E. faecium* resistance to linezolid, tigecycline, and daptomycin were analyzed by STATA software.

**Results:** The overall prevalence of antibiotic-resistant *E. faecalis* and *E. faecium* was reported to be 0.9 and 0.6%, respectively. *E. faecalis* and *E. faecium* were more resistant to the linezolid (2.2%) and daptomycin (9%), respectively. The prevalence of tigecyline-resistant *E. facium* (1%) strains was higher than *E. faecalis* strains (0.3%). Accordingly, the prevalence of linezolid-resistant *E. faecalis* was higher in Asia (2.8%), while linezolid-resistant *E. faecium* was higher in the America (3.4%). Regarding tigecycline-resistance, a higher prevalence of *E. faecalis* (0.4%) and *E. faecium* (3.9%) was reported in Europe.

**Conclusion:** In conclusion, this meta-analysis shows that there is an emerging resistance in *Enterococcus* strains. Despite the rising resistance of enterococci to antibiotics, our results demonstrate that tigecycline, daptomycin, and linezolid can still be used for the treatment of enterococcal infections worldwide.

## Introduction

Enterococci are known as opportunistic pathogens which are common inhabitants of the intestines of humans and animals (1, 2). They are not only a significant component of the commensal microbiota (3), but also are able to cause a wide variety of serious infections such as bacteremia, endocarditis, intra-abdominal and pelvic infection, and urinary tract infection (UTI) (4–6). The predominant species belonging to the genus *Enterococcus* in clinical specimens are *Enterococcus faecalis* (*E. faecalis*) and *Enterococcus faecium* (*E. faecium*). These two species are currently considered as the second and third most important nosocomial pathogens in the world (7, 8). Currently, expansion of antimicrobial resistance is one of the most important global concerns (9). Acquired resistance to several antimicrobial agents is more frequently observed in *E. faecium* than in *E. faecalis*. The World Health Organization (WHO) considered vancomycin-resistant *E. faecium* as a “high priority pathogen” urgently requiring new antibiotics for targeted treatment (5). *E. faecium* belongs to the “ESKAPE” pathogens, which includes six bacteria with growing multidrug resistance and virulence: *E. faecium, Staphylococcus aureus, Klebsiella pneumoniae, Acinetobacter baumannii, Pseudomonas aeruginosa*, and *Enterobacter* spp. These bacteria are responsible for majority of nosocomial infections and they can escape from the biocidal action of antimicrobial agents (10, 11). Resistance is associated with increased morbidity and mortality rates (12, 13). Vancomycin was considered as one of the last lines of treatment against multidrug-resistant bacteria. However, the emergence of Vancomycin-Resistant Enterococci (VRE) among hospitalized patients who had received long lasting courses of antibiotic treatment, posed a serious threat for other patients and health care professionals (14–17). More than 40% of *E. faecium* bloodstream isolates was resistant to vancomycin, and the prevalence of VRE was different in various countries (9-12.5%) (8, 18). Therefore, the emergence of VRE has prompted the use of novel and modified therapeutic agents including linezolid, daptomycin, and tigecycline, although resistance to those agents has already been reported in clinical settings (19, 20). Linezolid is the first US FDA approved oxazolidinone antibiotic and it has great therapeutic efficacy for severe infections caused by multidrug-resistant Gram-positive organisms including VRE (21–23). Mechanisms of linezolid resistance in *Enterococcus* spp. is often due to mutations in the 23S ribosomal RNA (rRNA) genes and ribosomal protein-coding regulatory genes such as *rplC, rplD*, and *rplV*, mutations leading to amino acid substitutions in several ribosomal proteins [L3, L4 and/or L22], and the acquisition of more generic resistance genes such as *cfr, cfr(B), poxtA*/*optrA* (24–26). In 2003, the FDA approved the therapeutic use of the bactericidal lipopeptide antibiotic daptomycin against complicated skin and soft tissue infections arising from a broad spectrum of Gram-positive bacteria, including VRE (27, 28). Emergence of daptomycin non-susceptibility in enterococci is associated with mutations in several genes including the stress-sensing response system YycFGHIJ and LiaFSR and alterations in phospholipid biosynthesis enzymes such as cardiolipin synthetase *cls* and glycerophosphoryl diester phosphodiesterase *gdpD* (29–31). A third antibiotic that was shown to be beneficial against VRE infection is the bacteriostatic tigecycline that blocks bacterial protein synthesis at the elongation stage (32). Mutations in various efflux pumps is the main mechanism that associated with tigecycline-resistance in the enterocooci. Other resistance-related mechanisms are deletions in ribosomal protein gene *rpsJ* and elimination of transcriptional regulation of the ribosomal protection protein (33, 34). In conclusion, several new treatment modalities for enterococcal infections have been introduced over the past decades and now is a good moment to define the emergence of resistance to these relatively novel agents used. In the present study, a systematic review and meta-analysis was conducted to define the current prevalence of resistance to linezolid, daptomycin, and tigecycline among *E. faecalis* and *E. faecium* strains isolated from human clinical specimens worldwide.

## Methods

### Search Strategy and Selection Criteria

We reviewed original research articles available in databases and published between 2000 and 2020. These databases include Medline (*via* PubMed), Embase, and Web of Science. We searched the databases on December 2020. *Enterococcus faecalis* or *E. faecalis OR Enterococcus faecium* or *E. faecium* and *linezolid* and *daptomycin* and *tigecycline* were the keywords used in our search strategy. The searches in this study selectively included articles published in the field of epidemiology of *E. faecalis* and *E. faecalis* strains isolated from human specimens and targeted to define the prevalence of Antibiotic-Resistant Enterococci (ARE), Linezolid-Resistant *E. faecalis* (LREF), Tigecycline-Resistant *E. faecalis* (TREF), Daptomycin-Resistant *E. faecalis* (DREF), Linezolid-Resistant *E. faecium* (LREFA), Tigecycline-Resistant *E. faecium* (TREFA), and Daptomycin-Resistant *E. faecium* (DREFA). It is worth noting that the latest version of the CLSI guideline (2020) stated that linezolid-resistance in *Enterococcus* spp. as determined by disk diffusion (DD) must be confirmed by an MIC-generating method. As a result, studies that have used only the DD method to determine susceptibility to linezolid were excluded from the present study. Other excluded studies were review articles, case reports, and publications on basic research of resistance mechanisms for the mentioned antibiotics. Still, the bibliographies of excluded literature were explored to recognize further studies.

### Inclusion and Exclusion Criteria

All studies on human clinical samples with complete information about the prevalence of ARE were evaluated. These data included prevalence or frequency of infection by *Enterococcus* spp. (*E. faecalis* and *E. faecium*), the country of origin, and the resistance assessment methods applied. The information presented in each study was evaluated using titles, abstracts, and, ultimately, the full text. Studies that qualified include original articles that provide sufficient information on the prevalence of ARE isolated from human specimens. Studies that used validated molecular techniques to diagnose antibiotic-resistant enterococci and presented data regarding the number of enrolled patients were all included. Excluded studies were those performed on non-human cases, studies that investigated other species of *Enterococcus* spp., covering other types of antibiotics, meta-analysis, systematic reviews and review articles, congress abstracts, and duplicate publications for the same investigation. Two authors separately reviewed inclusion and exclusion criteria and jointly selected appropriate articles.

### Data Extraction and Definitions

The following data were extracted from the studies that met the inclusion criteria: the first author's last name, study period, year of publication, country, numbers of antibiotic-resistant *E. faecalis* and *E. faecium* strains, source of samples, and detection techniques (including genotypic identification methods applied). In order to do more accurate data extraction, this was done by two independent individuals and eventually confirmed by another researcher. In order to reach consensus, the reviewers finally had a closing discussion.

### Quality Assessment

For all the studies which were entered in our searches based on the desired keywords, a quality evaluation (designed by the Joanna Briggs Institute) has been performed and only the studies with high-quality evaluation were selected for the final analysis (35).

### Meta-Analysis

STATA (version 14.0) software was used for data analysis. Two models were used to pool the obtained data: fixed effects model (FEM) (36) and a random effects model (REM) (37). Various statistical methods were used to assess statistical heterogeneity, and then the heterogeneity was evaluated by *Q*-test and I2 statistical methods (38). *P*-value <0.1 was regarded as statistically significant (38).

## Results

### Characteristics of Included Studies

**Figure 1:**
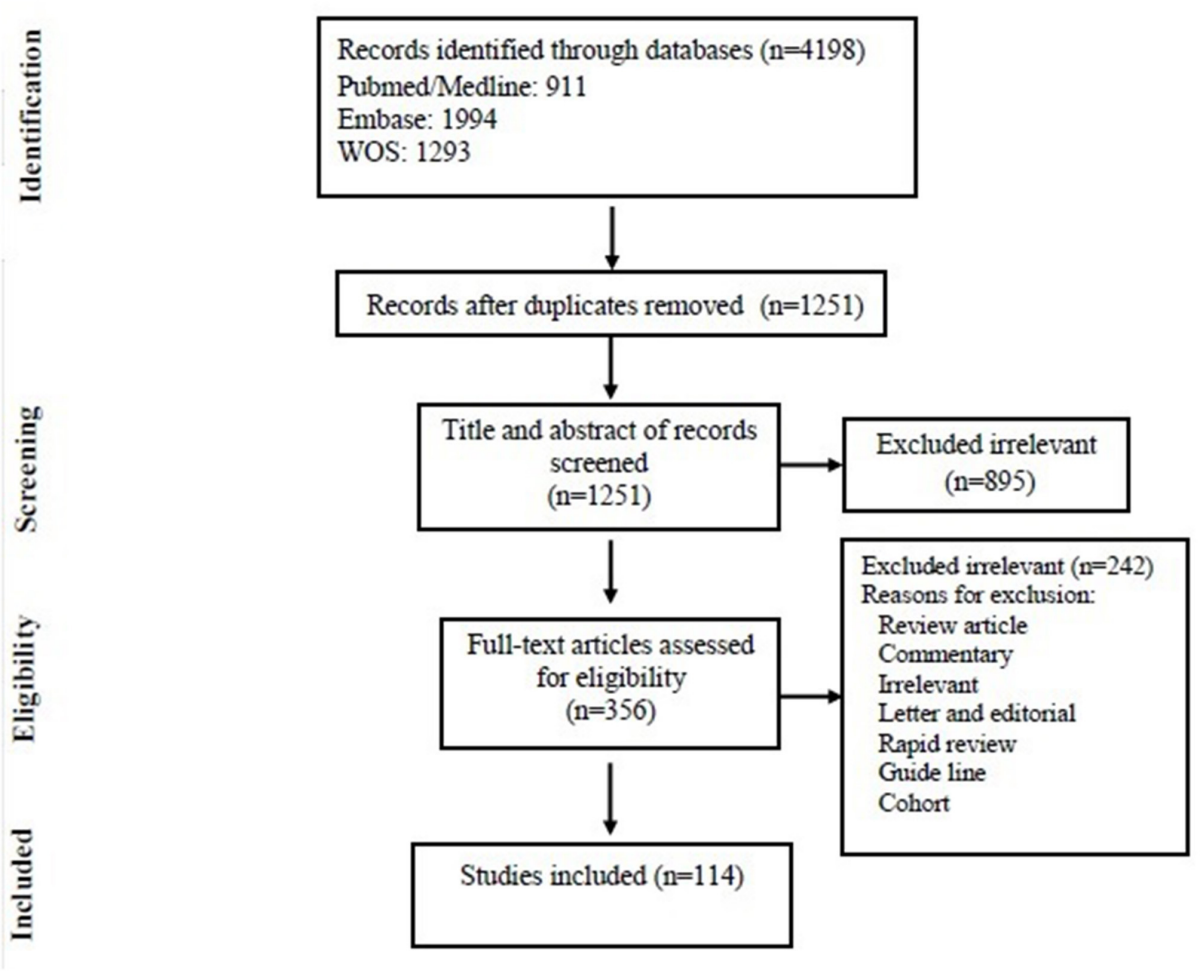
Flow chart of study selection for inclusion in the systematic review and meta-analysis.

Among 4,198 articles, which were selected after an initial review of electronic resources and databases, 2,947 duplicate articles were excluded and 1,251 unique articles remained. After the assessment of the title and abstract, 895 articles were excluded and 356 articles remained of which 242 were excluded upon full-text search (see Figure 1 for more detailed explanation). Finally, 114 articles met the inclusion criteria of this study and were selected for final statistical analysis. Out of these 114 studies, 40 articles (35.08%) were from the Asian continent, 28 Europe (24.56%), 45 from America (39.47%), and 1 (0.87%) from Africa. There were no studies from Oceania. Most of the articles reviewed in this study were from USA (40 articles) and China (25 articles), respectively. In Supplementary Tables 1–3, the main features of the 114 selected articles are summarized. Most of the studies included in this study were published between 2012 and 2020 (more than 50%).

### The Prevalence of the LREF, TREF, DREF, *E. faecalis, E. faecium*, LREFA, TREFA, and DREFA Among Clinical Isolates

As shown in **Table 3**, antibiotic resistance was reported for the three antibiotics linezolid, daptomycin, and tigecycline [0.7% (95% CI 0.4-1.1)] among 362,604 *Enterococcus* isolates. The prevalence of AREF and AREFA were reported as 0.9% (95% CI 0.4-1.1) among 180,917 and 0.6% (95% CI 0.1-1.0) among 181,687 isolates, respectively. As a result, based on the analysis of the included data, the AREF-associated resistance is more frequent than defined for the AREFA strains. The pooled prevalence of *E. faecalis* and *E. faecium* strains was reported at 47.6% (95% CI 43.8-51.3) among 6,036 isolates and 40.6% (95% CI 38.3-42.8) among 115,406 isolates, respectively. The results of AREF analysis showed that LREF isolates had a prevalence of 2.2% (95% CI 1.5-2.8) which was more prevalent than TREF and DREF strains. According to Table 1, the following can be concluded about AREFA strains: The resistance of these strains to linezolid at 1.1% (95% CI 0.3-1.9) was the most frequent and then there were tigecycline-resistance at 1.0% (95% CI 0.0-3.5) and daptomycin-resistance at 9.0% (95% CI 3.8-14.2), respectively.

**Table 1:** Prevalence of the LREF, TREF, DREF, *E. faecalis, E. faecium*, LREFA, TREFA, and DREFA based on the meta-analysis of the included studies.

**Isolate**	**Category**	**Subcategory**	**No. of studies**	**No. of strains**	**Prevalence (%) (95% CI)**	**I2**
ARE	Overall	ARE*/Enterococcus*	161	2,600/362,604	0.7 (0.4-1.1)	0.0%
AREF	Overall	AREF*/Enterococcus*	72	1,593/180,917	0.9 (0.4-1.4)	0.0%
AREFA	Overall	AREFA*/Enterococcus*	89	1,007/181,687	0.6 (0.1-1.0)	0.0%
LREF	Overall	LREF*/E. faecalis*	43	1,646/69,291	2.2 (1.5-2.8)	0.0%
TREF	Overall	TREF*/E. faecalis*	9	25/10,449	0.3 (0.0-2.2)	0.0%
DREF	Overall	DREF*/E. faecalis*	4	32/11,821	0.1 (0.0-0.3)	88.5%
*E. faecalis*	Overall	*E. faecalis/Enterococcus*	22	3,061/6,036	47.6 (43.8-51.3)	92.0%
*E. faecium*	Overall	*E. faecium/Enterococcus*	39	49,613/115,406	40.6 (38.3-42.8)	88.9%
DREFA	Overall	DREFA*/E. faecium*	11	295/7,226	9.0 (3.8-14.2)	62.5%
TREFA	Overall	TREFA*/E. faecium*	9	58/6,134	1.0 (0.0-3.5)	0.0%
LREFA	Overall	LREFA*/E. faecium*	51	574/52,605	1.1 (0.3-1.9)	0.0%

### The Prevalence of the LREF, TREF, DREF, *E. faecalis, E. faecium*, LREFA, TREFA, and DREFA Among Different Continents

**Figure 2:**
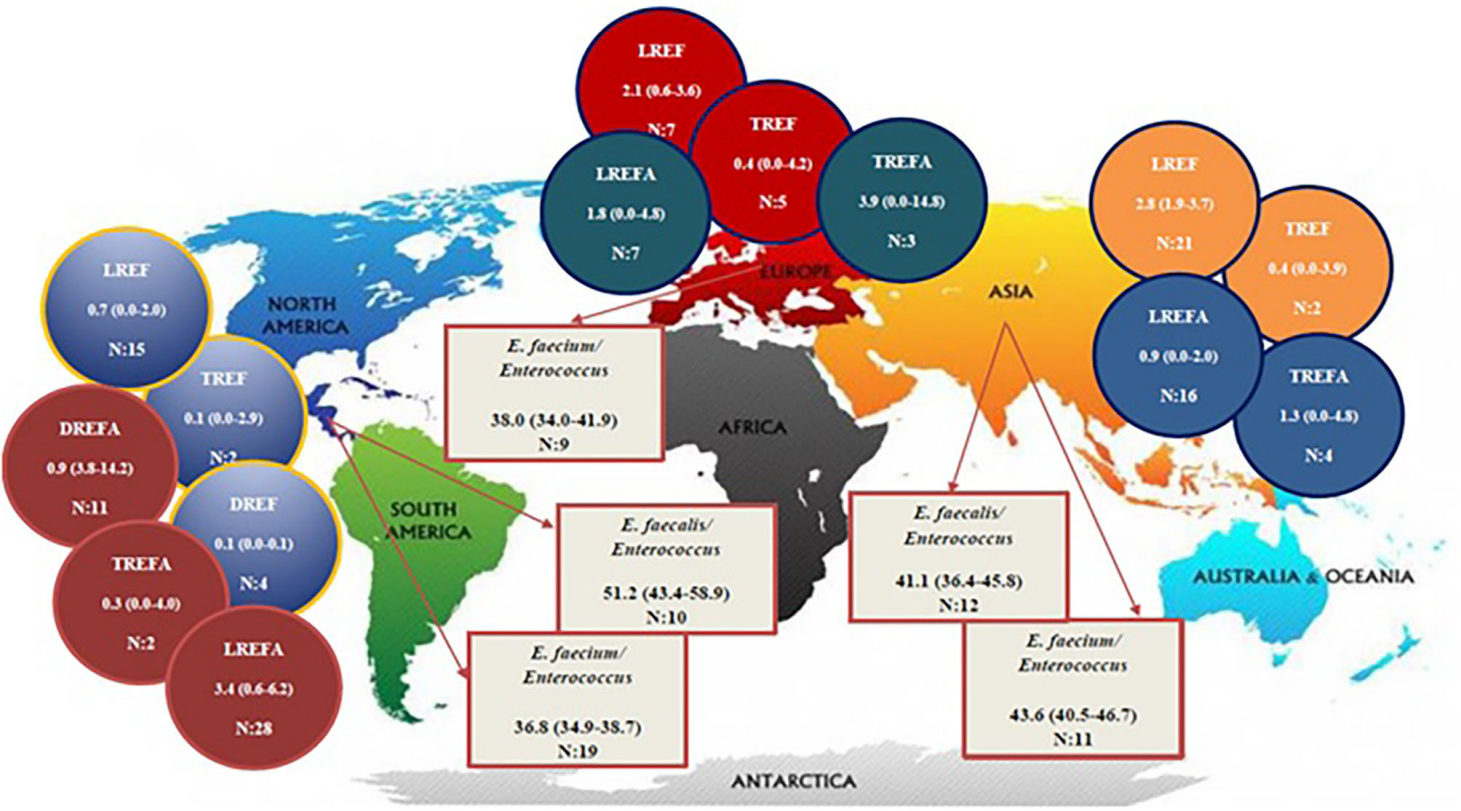
Distribution of the *LREF, TREF, DREF, LREFA, TREFA*, and *DREFA* among different continents.

Table 2 and Figure 2 show the prevalence of *E. faecalis* and *E. faecium* strains resistant to one of the antibiotics linezolid, daptomycin, and tigecycline in different continents. The prevalence of all *Enterococcus* strains were included in this study in the America and Asia were 51.2% (95% CI 43.4-58.9) and 41.1% (95% CI 36.4-45.8), respectively. Therefore, it can be concluded that the prevalence of *E. faecalis* strains that were included in our study in the America (1,908/3,436) is higher than in Asia (1,153/2,600). The results of this study also demonstrated that the prevalence of included *E. faecium* strains in this study was highest in Asia [43.6% (95% CI 40.5-46.7)] followed by Europe [38.0% (95% CI 34.0-41.9)] and America [36.8% (95% CI 34.9-38.7)]. Accordingly, the prevalence of LREF strains in Asia, Europe and the America were 2.8% (95% CI 1.9-3.7), 2.1% (95% CI 0.6-3.6), and 0.7% (95% CI 0.0-2.0), respectively. Regarding TREF strains, our analysis showed that the prevalence of these strains in Asia, Europe, and the America were 0.4% (95% CI 0.0-3.9, 0.4% (95% CI 0.0-4.2), and, somewhat lower, 0.1% (95% CI 0.0-2.9), respectively. As shown in Table 2, the prevalence of DREF strains in the America is reported to be 0.1% (CI 95% 0.0-0.1). This Table also reports the prevalence of *E. faecium* strains resistant to linezolid, daptomycin, and tigecycline on different continents. The prevalence of DREFA strains in the America was 9.0% (95% CI 3.8-222 14.2) among 7,226 isolates (Table 2). In addition, the resistance of TREFA strains in Asia, Europe, and America were 1.3% (95% CI 0.0-4.8), 3.9% (95% CI 0.0-14.8), and 0.3% (95% CI 0.0-4.0), respectively. It is reported that the prevalence of these strains in Europe (27/2,048) was higher than in Asia (22/1,310) and in the United States (9/2,776). These results show that the prevalence of these strains in Europe (27/2,048) was higher than Asia (22/1,310) and the United States (9/2,776). The results of this study also showed that the prevalence of LREFA strains in Asia, Europe, and the America were 0.9% (95% CI 0.0-2.0), 1.8% (95% CI 0.0-4.8), and 3.4%, (95% CI 0.6-6.2), respectively. Accordingly, the prevalence of these strains in the United States was 1.37% (233/16,929) which is higher than in Europe with 1.21% (45/3,694) and in Asia with 0.92% (296/31,982).

**Table 2:** Prevalence of the LREF, TREF, DREF, *E. faecalis, E. faecium*, LREFA, TREFA, and DREFA based on continents.

**Isolate**	**Category**	**Subcategory**	**No. of studies**	**No. of strains**	**Prevalence (%) (95% CI)**	**I2**
LREF	Overall	LREF*/E. faecalis*	**43**	**1,646/69,291**	**2.2 (1.5-2.8)**	**0.0%**
	Continent	Asia	21	1,200/37,485	2.8 (1.9-3.7)	0.0%
		Europe	7	283/9,283	2.1 (0.6-3.6)	0.0%
		America	15	163/22,523	0.7 (0.0-2.0)	0.0%
TREF	Overall	TREF*/E. faecalis*	**9**	**25/10,449**	**0.3 (0.0-2.2)**	**0.0%**
	Continent	Asia	2	6/118	0.4 (0.0-3.9)	27.3%
		Europe	5	13/5,498	0.4 (0.0-4.2)	0.0%
		America	2	6/4,833	0.1 (0.0-2.9)	0.0%
DREF	Overall	DREF*/E. faecalis*	**4**	**32/11,821**	**0.1 (0.0-0.1)**	**88.5%**
	Continent	America	4	32/11,821	0.1 (0.0-0.1)	88.5%
*E. faecalis*	Overall	*E. faecalis/Enterococcus*	**22**	**3,061/6,036**	**47.6 (43.8-51.3)**	**92.0%**
	Continent	Asia	12	1,153/2,600	41.1 (36.4-45.8)	87.0%
		America	10	1,908/3,436	51.2 (43.4-58.9)	94.7%
*E. faecium*	Overall	*E. faecium/Enterococcus*	**39**	**49,613/115,406**	**40.6 (38.3-42.8)**	**88.9%**
	Continent	Asia	11	32,352/68,125	43.6 (40.5-46.7)	91.7%
		Europe	9	2,492/7,084	38.0 (34.0-41.9)	68.4%
		America	19	14,769/40,197	36.8 (34.9-38.7)	71.3%
DREFA	Overall	DREFA*/E. faecium*	**11**	**295/7,226**	**9.0 (3.8-14.2)**	**62.5%**
	Continent	America	11	295/7,226	9.0 (3.8-14.2)	62.5%
TREFA	Overall	TREFA*/E. faecium*	**9**	**58/6,134**	**1.0 (0.0-3.5)**	**0.0%**
	Continent	Asia	4	22/1,310	1.3 (0.0-4.8)	0.0%
		Europe	3	27/2,048	3.9 (0.0-14.8)	5.2%
		America	2	9/2,776	0.3 (0.0-4.0)	0.0%
LREFA	Overall	LREFA*/E. faecium*	**51**	**574/52,605**	**1.1 (0.3-1.9)**	**0.0%**
	Continent	Asia	16	296/31,982	0.9 (0.0-2.0)	0.0%
		Europe	7	45/3,694	1.8 (0.0-4.8)	0.0%
		America	28	233/16,929	3.4 (0.6-6.2)	57.5%

### The Prevalence of the LREF, TREF, DREF, *E. faecalis, E. faecium*, LREFA, TREFA, and DREFA in Different Regions of the World

The prevalence of *E. faecalis* and *E. faecium* strains resistant to the three studied antibiotics is shown in Table 3. The prevalence of *E. faecalis* strains included in this study in the United States, China, and Turkey were 53.2% (95% CI 45.5-60.8), 44.3% (95% CI 37.5-50.9), and 39.1% (95% CI 26.2-51.9), respectively. In addition, the prevalence of *E. faecium* strains included in our study in China, UK, USA, and Italy were 50.1% (95% CI 47.7-52.5), 48.5% (95% CI 38.9-58.1), 37.0% (95% CI 35.2-38.9), and 34.9% (95% CI 33.0-36.8), respectively. Accordingly, China with 47.48% (32,352/68,125) had the highest prevalence of *E. faecium* strains in the study. LREF is most prevalent in Turkey 7.1% (95% CI 0.0-21.5), Germany 4.6% (95% CI 2.1-7.0), and China 3.2% (95% CI 2.2-4.2). The prevalence of TREFA strains was similar to that of LREF. As shown in this Table, DREF strains have been reported in the United States at a rate of 0.1% (95% CI 0.0-0.1). The prevalence of DREFA strains has also been reported in the United States at a rate of 9.0% (95% CI 3.8-14.2). As shown in Table 3, the prevalence of LREFA strains in China, Germany, India, Italy, and the United States were 0.9% (95% CI 0.0-2.0), 2.1% (95% CI 0.0-9.5), 6.0% (95% CI 0.0-26.7), 1.1% (95% CI 0.0-4.6), and 3.6% (95% CI 0.5-6.7), respectively, indicating a high prevalence of these strains in the United States compared to other countries.

**Table 3:** Prevalence of the LREF, TREF, DREF, *E. faecalis, E. faecium*, LREFA, TREFA, and DREFA based on countries.

**Isolate**	**Category**	**Subcategory**	**No. of studies**	**No. of strains**	**Prevalence (%) (95% CI)**	**I2**
LREF	Overall	LREF*/E. faecalis*	**43**	**1,646/69,291**	**2.2 (1.5-2.8)**	**0.0%**
	Country	China	14	1,179/36,870	3.2 (2.2**-**4.2)	0.0%
		Germany	4	278/6,125	4.6 2.1**-**7.0)	0.0%
		India	4	9/443	2.0 (0.0**-**11.3)	0.0%
		Spain	3	5/3,158	0.2 (0.0**-**3.6)	0.0%
		Turkey	3	12/172	7.1 (0.0**-**21.5)	0.0%
		USA	15	163/22,523	0.8 (0.0**-**2.1)	0.0%
TREF	Overall	TREF*/E. faecalis*	**9**	**25/10,449**	**0.3 (0.0-2.2)**	**0.0%**
	Country	*	*	*	*	*
DREF	Overall	DREF*/E. faecalis*	**4**	**32/11,821**	**0.1 (0.0-0.1)**	**88.5%**
	Country	USA	4	32/11,821	0.1 (0.0**-**0.1)	88.5%
*E. faecalis*	Overall	*E. faecalis/Enterococcus*	**22**	**3,061/6,036**	**47.6 (43.8-51.3)**	**92.0%**
	Country	China	8	1,006/2,240	44.3 (37.5**-**50.9)	89.8%
		Turkey	4	147/360	39.1 (26.2**-**51.9)	85.3%
		USA	10	1,908/3,436	53.2 (45.5**-**60.8)	93.9%
*E. faecium*	Overall	*E. faecium/Enterococcus*	**39**	**49,613/115,406**	**40.6 (38.3-42.8)**	**88.9%**
	Country	China	11	32,352/68,125	50.1 (47.7**-**52.5)	73.0%
		Italy	6	2,392/6,870	34.9 (33.0**-**36.8)	0.0%
		UK	3	100/214	48.5 (38.9**-**58.1)	46.5%
		USA	19	14,769/40,197	37.0 (35.2**-**38.9)	71.4%
DREFA	Overall	DREFA*/E. faecium*	**11**	**295/7,226**	**9.0 (3.8-14.2)**	**62.5%**
	Country	USA	11	295/7,226	9.0 (3.8**-**14.2)	62.5%
TREFA	Overall	TREFA*/E. faecium*	**9**	**58/6,134**	**1.0 (0.0-3.5)**	**0.0%**
	Country	*	*	*	*	*
LREFA	Overall	LREFA*/E. faecium*	**51**	**574/52,605**	**1.1 (0.3-1.9)**	**0.0%**
	Country	China	13	291/31,898	0.9 (0.0**-**2.0)	0.0%
		Germany	4	31/3,000	2.1 (0.0**-**9.5)	0.0%
		India	3	5/84	6.0 (0.0**-**26.7)	0.0%
		Italy	3	14/694	1.1 (0.0**-**4.6)	0.0%
		USA	28	233/16,929	3.6 (0.5**-**6.7)	63.9%

Because the countries that reported the prevalence of TREF strains were not large enough to be sub grouped, the prevalence of these strains in the countries was marked with *.

## Discussion

VRE are considered as an important nosocomial pathogen among the hospitalized patients and they are known to cause life threatening infections in humans. Various factors are predictive of VRE infections. These include long-term hospitalization, extensive use of antibiotics, and increased occupancy rate especially with malignancy in the intensive care unit (ICU) (39, 40). Surviving under harsh conditions and outstanding adaptation to environmental conditions render enterococci into a significant reservoir for the transmission and spread of antibiotic-resistance determinants (41). Higher mortality rates, extended length of hospital stay, and higher treatment costs are the consequences of VRE bacteremia (17). Furthermore, to limit mortality, early administration of appropriate antibiotic therapy is essential (42). According to studies, most *Enterococcus* spp. isolates from human infections belong to *E. faecalis* (43). In our systematic study and meta-analysis, *E. faecalis* (47.6%) was identified to be the most dominant species than *E. faecium* (40.6%). The rate of antibiotic-resistance was higher in *E. faecalis*. While daptomycin is more active against *E. faecalis*, linezolid and tigecycline can be used at low concentrations for both *E. faecalis* and *E. faecium* (44). Daptomycin has received FDA approval against complicated skin and soft-tissue infections due to VRE and MRSA in 2003 (45). The results of the present study show that daptomycin had the best remaining inhibitory effect on *E. faecalis* with a resistance rate of 0.1%, which is in accordance with previous studies showed that most enterococcal isolates (>99.8%) are susceptible to daptomycin on a worldwide scale. As mentioned above the potential reason is its higher activity levels against *E. faecalis*. It seems that prior but insufficient daptomycin exposure can be related to the occurrence of daptomycin-resistance among enterococci (8). Furthermore, statistical analysis demonstrated that DREFA were mostly isolated from bloodstream infections (BSI) in the United States. Despite of lack of FDA confirmation, daptomycin is used frequently for VRE-BSI. The results of previous studies showed that linezolid and daptomycin have similar results for the treatment of VRE-BSI. Since linezolid is bacteriostatic, and daptomycin is bactericidal, it is assumed that in the immunocompromised patients, daptomycin, achieves superior clinical and microbiologic response rates (42). However, spontaneous resistance to daptomycin seems to arise rarely (28, 46–49). Previous studies have reported that pulmonary surfactants have inhibitory effect on daptomycin, preventing this antibiotic to be effective on BSI following the lower respiratory tract infection and pneumonia (50). Furthermore, linezolid may provide a better therapeutic effect against BSI (51). Hence, daptomycin MICs should be screened in more detail to prevent treatment failure and the emergence of resistance. Studies showed *in vitro* synergistic interactions between daptomycin and various β-lactams in susceptible and non-susceptible enterococcal strains (52–54). This finding can be because of increased susceptibility of bacteria or enhanced surface binding by daptomycin and β-lactams (52). Linezolid is one of the last lines of defense to treat skin and lower respiratory tract infections. It inhibits bacterial growth by suppressing protein synthesis in bacteria (55). Several nosocomial case reports and outbreaks have been reported for both vancomycin-susceptible and vancomycin-resistant *E. faecium* and *E. faecalis* (8). *E. faecalis* strains have the highest resistance rate to linezolid (2.2%), while this rate is reported to be 1.1% among *E. faecium* strains. Africa and the USA had the highest rate of resistance from LREF and LREFA isolates with 13.9 and 3.4%, respectively. Nevertheless, differences in resistance rates depend on the various geographical regions and the species analyzed. There have been different resistance percentages reported from China, Denmark, Spain, Germany, etc. (8). Linezolid was introduced in the year 2000 as a therapeutic agent for infections caused by resistant Gram-positive cocci. Regrettably, widespread use of this antibiotic over the last 20 years has resulted in an emerging of linezolid-resistant VRE in 2001 and increasing of these strains especially in hospitals (42, 56). The Infectious Diseases Society of America (IDSA) recommended linezolid for VRE intravascular catheter-related bacteremia but significant side effects such as myelotoxicity led to its limited use, especially among immunocompromised patients (57). The multidrug-resistance gene *cfr* is one of the linezolid-resistance mechanisms in enterococci (8). This plasmid-defined mechanism can be occurring even across bacterial species and genera, as it is similar in *Staphylococcus* spp. and *Enterococcus* spp. This gene was first isolated from *Staphylococcus sciuri* of animal origin. Among enterococci it was first reported from an *E. faecalis* strain of the animal origin that can lead to resistance to at least five classes of antibiotics including linezolid (8). Tigecycline, a bacteriostatic agent which has a broad spectrum therapeutic effect against MDR Gram-positive bacteria including VRE and MRSA in addition to β-lactamase–producing Enterobacterales and anaerobes (58). Tigecycline is a potential treatment choice for complicated soft tissue and intra-abdominal infection, conversely, due to insufficient serum concentration, the use of it is restricted to bloodstream infections (59, 60). The emergence of tigecycline-resistant enterococci is increasing (61). According to the results obtained in this study, tigecycline has the best performance against *E. faecium* (compare to daptomycin and almost equal to linezolid); however, 0.3% of *E. faecalis* and 1% of *E. faecium* were resistance to this antibiotic. Resistance reports to tigecycline were gathered from different continents with the highest rate in *E. faecium* (3.9%) in Europe and *E. faecalis* (0.4%) in Europe and Asia. Recent reports of tigecycline susceptibility showed no increase in resistance among Gram-positive clinical isolates in the world over time. In accordance with our study, the results of another study revealed that 99.7% of enterococcal isolates (14,615 *E. faecalis* and 6,167 *E. faecium*) were susceptible (62). Moreover, most of the studies reported <1% tigecycline-resistance and that depended on the various geographical regions and the analyzed species (8). Compared to our previous study (63), the percentage of resistance to linezolid, tigecycline and daptomycin is higher in *Enterococcus* spp. than in *Staphylococcus* spp. in the world. It should be noted that a higher level of resistance to the mentioned antibiotics in some parts of the world may not imply a higher resistance to these antibiotics in these areas and may be consistent with regular microbial susceptibility testing programs or the number of studies and the studied isolates which was carried out in these countries. Therefore, by performing regular surveillance programs, the accurate prevalence of antibiotic-resistance can be determined effectively. It should be noted that there are several limitations to our study. First, only published full-text research articles were evaluated in our study. Second, only the studies on clinical enterococci isolates were evaluated and other studies on environmental samples were excluded. Third, since there is insufficient information from many countries, we were not able to provide a truly global representation. Fourth, the failure to differentiate the clinical samples, which eventually did not conclude the prevalence of *Enterococcus* in various infections. Fifth, as some countries do not systematically monitor resistant *E. faecalis* and *E. faecium*, the number of reported antibiotic-resistant enterococci isolated from clinical specimens may not be realistic and it can be less than the actual amount. In some cases, bacteria may colonize in the patient's body without clinical signs. Also, failure to comply with the guidelines on the isolation and identification of bacteria resistant to antimicrobial agents can lead to unrealistic results. Sixth, studies indicate that infections caused by antibiotic-resistance *Enterococcus* spp. may be associated with mortality. Seventh, we did not distinguish according to the technology used for resistance assessments. Different methods can lead to different results and in this study; we simply merged all data obtained by various methods. Despite the importance of this issue, studies on the impact of antibiotic-resistance *Enterococcus* on mortality are rare and the necessity of such studies is clear today.

## Conclusion

The present study reveals higher rates of resistance to daptomycin and tigecycline among *E. faecium* strains, whilst resistance to linezolid was higher in *E. faecalis*. By the way, our results show tigecycline, linezolid, and daptomycin still remain active against enterococcal isolates and can be used for the treatment of enterococcal infections worldwide. Obviously, monitoring of the rising resistance of VRE to these agents, appropriate antibiotic-resistance testing programs, and adequate antibiotic stewardship are extremely important in the successful reduction of resistance to the mentioned antibiotics, especially in VRE isolates.

## Data Availability Statement

The original contributions presented in the study are included in the article/Supplementary Material, further inquiries can be directed to the corresponding author/s.

## Author Contributions

DD-S and MD designed the study, wrote and edited the manuscript, separately reviewed inclusion and exclusion criteria, and assumed overall responsibility for the accuracy and integrity of the manuscript. BH conducted the search strategy. PS, NazB, NK-D, NarB, and BH performed the data extraction. MD carried out the statistical analysis. AB did critical editing and revising of the text. All authors contributed to the article and approved the submitted version.

## Conflict of Interest

AB is an employee of bioMérieux, a French company developing diagnostic tools for infectious diseases. The company has had no influence on the design and execution of the current study. The remaining authors declare that the research was conducted in the absence of any commercial or financial relationships that could be construed as a potential conflict of interest.

## Publisher's Note

All claims expressed in this article are solely those of the authors and do not necessarily represent those of their affiliated organizations, or those of the publisher, the editors and the reviewers. Any product that may be evaluated in this article, or claim that may be made by its manufacturer, is not guaranteed or endorsed by the publisher.

## References

[1] Tollu G Ekin IH. Biotyping and antimicrobial susceptibility of *Enterococcus faecalis* and *E. faecium* isolated from urine and stool samples. Jundishapur J Microbiol. (2020) 13:e105136. 10.5812/jjm.105136

[2] Stagliano DR Susi A Adams DJ Nylund CM. Epidemiology and outcomes of vancomycin-resistant enterococcus infections in the US Military Health System. Mil Med. (2021) 186(Supplement_1):100–7. 10.1093/milmed/usaa229 33499465

[3] Korpela K Blakstad EW Moltu SJ Strømmen K Nakstad B Rønnestad AE. Intestinal microbiota development and gestational age in preterm neonates. Sci Rep. (2018) 8:1–9. 10.1038/s41598-018-20827-x 29410448 PMC5802739

[4] van Harten RM Willems RJ Martin NI Hendrickx AP. Multidrug-resistant enterococcal infections: new compounds, novel antimicrobial therapies? Trends Microbiol. (2017) 25:467–79. 10.1016/j.tim.2017.01.004 28209400

[5] Contreras GA Munita JM Arias CA. Novel strategies for the management of vancomycin-resistant enterococcal infections. Curr Infect Dis Rep. (2019) 21:1–8. 10.1007/s11908-019-0680-y 31119397

[6] Linden PK. Treatment options for vancomycin-resistant enterococcal infections. Drugs. (2002) 62:425–41. 10.2165/00003495-200262030-00002 11827558

[7] Rodríguez-Noriega E Hernández-Morfin N Garza-Gonzalez E Bocanegra-Ibarias P Flores-Treviño S Esparza-Ahumada S. Risk factors and outcome associated with the acquisition of linezolid-resistant *Enterococcus faecalis*. J Glob Antimicrob Resist. (2020) 21:405–9. 10.1016/j.jgar.2020.01.010 32004724

[8] Bender JK Cattoir V Hegstad K Sadowy E Coque TM Westh H. Update on prevalence and mechanisms of resistance to linezolid, tigecycline and daptomycin in enterococci in Europe: towards a common nomenclature. Drug Resist Updat. (2018) 40:25–39. 10.1016/j.drup.2018.10.002 30447411

[9] Prestinaci F Pezzotti P Pantosti A. Antimicrobial resistance: a global multifaceted phenomenon. Pathog Glob Health. (2015) 109:309–18. 10.1179/2047773215Y.0000000030 26343252 PMC4768623

[10] Mulani MS Kamble EE Kumkar SN Tawre MS Pardesi KR. Emerging strategies to combat ESKAPE pathogens in the era of antimicrobial resistance: a review. Front Microbiol. (2019) 10:539. 10.3389/fmicb.2019.00539 30988669 PMC6452778

[11] Hasani A Purmohammad A Ahangarzadeh Rezaee M Hasani A Dadashi M. Integron-mediated multidrug and quinolone resistance in extended-spectrum β-lactamase-producing *Escherichia coli* and *Klebsiella pneumoniae*. Arch Pediatr Infect Dis. (2017) 5:e36616. 10.5812/pedinfect.36616

[12] O'Neil J. Tackling drug-resistant infections globally: final report and recommendations. (2016). Available online at: https://amr-review.org/sites/default/files/160518_Final%20paper_with%20cover.pdf

[13] Shrivastava SR Shrivastava PS Ramasamy J. World health organization releases global priority list of antibiotic-resistant bacteria to guide research, discovery, and development of new antibiotics. J Med Soc. (2018) 32:76. 10.4103/jms.jms_25_17

[14] Sarathy MV Balaji S Rao TJM. Enterococcal Infections and Drug Resistance Mechanisms. Model Organisms for Microbial Pathogenesis, Biofilm Formation and Antimicrobial Drug Discovery. Singapore: Springer Nature Singapore Pte Ltd. (2020). p. 131–58.

[15] Lund LC Holzknecht BJ Justesen US. Treatment of vancomycin-resistant enterococcal infections. Ugeskr Laeger. (2018) 180:V07170530. 29690991

[16] DiazGranados CA Zimmer SM Mitchel K Jernigan JA. Comparison of mortality associated with vancomycin-resistant and vancomycin-susceptible enterococcal bloodstream infections: a meta-analysis. Clin Infect Dis. (2005) 41:327–33. 10.1086/430909 16007529

[17] Hemapanpairoa J Changpradub D Thunyaharn S Santimaleeworagun W. Vancomycin-resistant enterococcal infection in a Thai university hospital: clinical characteristics, treatment outcomes, and synergistic effect. Infect Drug Resist. (2019) 12:2049. 10.2147/IDR.S208298 31372012 PMC6628965

[18] Emaneini M Hosseinkhani F Jabalameli F Nasiri MJ Dadashi M Pouriran R. Prevalence of vancomycin-resistant Enterococcus in Iran: a systematic review and meta-analysis. Eur J Clin Microbiol Infect Dis. (2016) 35:1387–92. 10.1007/s10096-016-2702-0 27344575

[19] Zaheer R Cook SR Barbieri R Goji N Cameron A Petkau A. Surveillance of *Enterococcus spp*. reveals distinct species and antimicrobial resistance diversity across a One-Health continuum. Sci Rep. (2020) 10:1–16. 10.1038/s41598-020-61002-5 32127598 PMC7054549

[20] Mendes RE Castanheira M Farrell DJ Flamm RK Sader HS Jones RN. Longitudinal (2001–14) analysis of Enterococci and VRE causing invasive infections in European and US hospitals, including a contemporary (2010–13) analysis of oritavancin *in vitro* potency. J Antimicrob Chemother. (2016) 71:3453–8. 10.1093/jac/dkw319 27609052

[21] Sadowy E. Linezolid resistance genes and genetic elements enhancing their dissemination in *Enterococci* and *Streptococci*. Plasmid. (2018) 99:89–98. 10.1016/j.plasmid.2018.09.011 30253132

[22] Bi R Qin T Fan W Ma P Gu B. The emerging problem of linezolid-resistant enterococci. J Glob Antimicrob Resist. (2018) 13:11–9. 10.1016/j.jgar.2017.10.018 29101082

[23] Wang J-L Hsueh P-R. Therapeutic options for infections due to vancomycin-resistant enterococci. Expert Opin Pharmacother. (2009) 10:785–96. 10.1517/14656560902811811 19351228

[24] Chen Q Yin D Li P Guo Y Ming D Lin Y. First report Cfr and OptrA Co-harboring Linezolid-resistant *Enterococcus faecalis* in China. Infect Drug Resist. (2020) 13:3919. 10.2147/IDR.S270701 33173316 PMC7646505

[25] Tsilipounidaki K Gerontopoulos A Papagiannitsis C Petinaki E. First detection of an optrA-positive, linezolid-resistant ST16 *Enterococcus faecalis* from human in Greece. New Microbes New Infect. (2019) 29:100515. 10.1016/j.nmni.2019.01.010 30899521 PMC6406052

[26] Liu Y Wang Y Schwarz S Li Y Shen Z Zhang Q. Transferable multiresistance plasmids carrying cfr in *Enterococcus spp*. from swine and farm environment. Antimicrob Agents Chemother. (2013) 57:42–8. 10.1128/AAC.01605-12 23070165 PMC3535926

[27] Turnidge J Kahlmeter G Cantón R MacGowan A Giske C Testing ECoAS. Daptomycin in the treatment of Enterococcal bloodstream infections and endocarditis: a EUCAST position paper. Clin Microbiol Infect. (2020) 26:1039–43. 10.1016/j.cmi.2020.04.027 32353412

[28] Arbeit RD Maki D Tally FP Campanaro E Eisenstein BI 98-01 D. The safety and efficacy of daptomycin for the treatment of complicated skin and skin-structure infections. Clin Infect Dis. (2004) 38:1673–81. 10.1086/420818 15227611

[29] Kelesidis T Humphries R Uslan DZ Pegues DA. Daptomycin nonsusceptible enterococci: an emerging challenge for clinicians. Clin Infect Dis. (2011) 52:228–34. 10.1093/cid/ciq113 21288849 PMC8483151

[30] Munita JM Panesso D Diaz L Tran TT Reyes J Wanger A. Correlation between mutations in liaFSR of *Enterococcus faecium* and MIC of daptomycin: revisiting daptomycin breakpoints. Antimicrob Agents Chemother. (2012) 56:4354–9. 10.1128/AAC.00509-12 22664970 PMC3421602

[31] Humphries RM Pollett S Sakoulas G. A current perspective on daptomycin for the clinical microbiologist. Clin Microbiol Rev. (2013) 26:759–80. 10.1128/CMR.00030-13 24092854 PMC3811228

[32] Ullah H Ali S. Classification of anti-bacterial agents and their functions. Antibacterial Agents. (2017) 10:1–10. 10.5772/intechopen.68695

[33] Levitus M Rewane A Perera TB. Vancomycin-Resistant Enterococci (VRE) StatPearls Publishing LLC (2020). 30020605

[34] Riccardi N Monticelli J Antonello RM Di Lallo G Frezza D Luzzati R. Therapeutic options for infections due to vanB genotype vancomycin-resistant *Enterococci*. Microb Drug Resist. (2020) 27:536–45. 10.1089/mdr.2020.0171 32799629

[35] Institute J. Joanna Briggs Institute Reviewers' Manual. The Joanna Briggs Institute South Australia (2014).

[36] Mantel N Haenszel W. Statistical aspects of the analysis of data from retrospective studies of disease. J Natl Cancer Inst. (1959) 22:719–48. 13655060

[37] DerSimonian R Laird N. Meta-analysis in clinical trials. Control Clin Trials. (1986) 7:177–88. 10.1016/0197-2456(86)90046-2 3802833

[38] Higgins JP Thompson SG. Quantifying heterogeneity in a meta-analysis. Stat Med. (2002) 21:1539–58. 10.1002/sim.1186 12111919

[39] Orababa OQ Soriwei JD Akinsuyi SO Essiet UU Solesi OM. A systematic review and meta-analysis on the prevalence of vancomycin-resistant enterococci (VRE) among Nigerians. Porto Biomed J. (2021) 6:e125. 10.1097/j.pbj.0000000000000125 33884321 PMC8055482

[40] Prematunge C MacDougall C Johnstone J Adomako K Lam F Robertson J. VRE and VSE bacteremia outcomes in the era of effective VRE therapy: a systematic review and meta-analysis. Infect Control Hosp Epidemiol. (2016) 37:26–35. 10.1017/ice.2015.228 26434609 PMC4707508

[41] Arias CA Contreras GA Murray BE. Management of multidrug-resistant enterococcal infections. Clin Microbiol Infect. (2010) 16:555–62. 10.1111/j.1469-0691.2010.03214.x 20569266 PMC3686902

[42] Twilla JD Finch CK Usery JB Gelfand MS Hudson JQ Broyles JE. Vancomycin-resistant *Enterococcus bacteremia*: an evaluation of treatment with linezolid or daptomycin. J Hosp Med. (2012) 7:243–8. 10.1002/jhm.994 22076962

[43] Sanlibaba P Senturk E. Prevalence, characterization and antibiotic resistance of enterococci from traditional cheeses in Turkey. Int J Food Properties. (2018) 21:1955–63. 10.1080/10942912.2018.1489413

[44] Maraki S Samonis G Dimopoulou D Mantadakis E. Susceptibility of glycopeptide-resistant Enterococci to linezolid, quinupristin/dalfopristin, tigecycline and daptomycin in a Tertiary Greek Hospital. Infect Chemother. (2014) 46:253. 10.3947/ic.2014.46.4.253 25566405 PMC4285005

[45] Sader H Farrell D Flamm R Jones R. Daptomycin activity tested against 164 457 bacterial isolates from hospitalised patients: Summary of 8 years of a Worldwide Surveillance Programme (2005-2012). Int J Antimicrob Agents. (2014) 43:465–9. 10.1016/j.ijantimicag.2014.01.018 24636430

[46] Balli EP Venetis CA Miyakis S. Systematic review and meta-analysis of linezolid versus daptomycin for treatment of vancomycin-resistant *Enterococcal bacteremia*. Antimicrob Agents Chemother. (2014) 58:734–9. 10.1128/AAC.01289-13 24247127 PMC3910884

[47] Chuang Y-C Lin H-Y Chen P-Y Lin C-Y Wang J-T Chen Y-C. Effect of daptomycin dose on the outcome of vancomycin-resistant, daptomycin-susceptible *Enterococcus faecium* Bacteremia. Clin Infect Dis. (2017) 64:1026–34. 10.1093/cid/cix024 28329222

[48] Narayanan N Rai R Vaidya P Desai A Bhowmick T Weinstein MP. Comparison of linezolid and daptomycin for the treatment of vancomycin-resistant enterococcal bacteremia. Ther Adv Infect Dis. (2019) 6:2049936119828964. 10.1177/2049936119828964 30792858 PMC6376491

[49] Silverman JA Oliver N Andrew T Li T. Resistance studies with daptomycin. Antimicrob Agents Chemother. (2001) 45:1799–802. 10.1128/AAC.45.6.1799-1802.2001 11353628 PMC90548

[50] Ye Y Xia Z Zhang D Sheng Z Zhang P Zhu H. Multifunctional pharmaceutical effects of the antibiotic daptomycin. Biomed Res Int. (2019) 2019:8609218. 10.1155/2019/8609218 31263709 PMC6556800

[51] Zasowski EJ Trinh TD Claeys KC Casapao AM Sabagha N Lagnf AM. Multicenter observational study of Ceftaroline fosamil for methicillin-resistant *Staphylococcus aureus* bloodstream infections. Antimicrob Agents Chemother. (2017) 61:e02015–16. 10.1128/AAC.02015-16 27895012 PMC5278749

[52] Avery LM Kuti JL Weisser M Egli A Rybak MJ Zasowski EJ. Pharmacodynamics of daptomycin in combination with other antibiotics for the treatment of *Enterococcal bacteraemia*. Int J Antimicrob Agents. (2019) 54:346–50. 10.1016/j.ijantimicag.2019.07.002 31284042

[53] Campeau SA Schuetz AN Kohner P Arias CA Hemarajata P Bard JD. Variability of daptomycin MIC values for *Enterococcus faecium* when measured by reference broth microdilution and gradient diffusion tests. Antimicrob Agents Chemother. (2018) 62:e00745–18. 10.1128/AAC.00745-18 29941639 PMC6125562

[54] Avery LM Kuti JL Weisser M Egli A Rybak MJ Zasowski EJ. Pharmacodynamic analysis of daptomycin-treated *Enterococcal bacteremia*: it is time to change the breakpoint. Clin Infect Dis. (2019) 68:1650–7. 10.1093/cid/ciy749 30188976 PMC6938208

[55] Leong HN Kurup A Tan MY Kwa ALH Liau KH Wilcox MH. Management of complicated skin and soft tissue infections with a special focus on the role of newer antibiotics. Infect Drug Resist. (2018) 11:1959. 10.2147/IDR.S172366 30464538 PMC6208867

[56] Stefani S Bongiorno D Mongelli G Campanile F. Linezolid resistance in Staphylococci. Pharmaceuticals. (2010) 3:1988–2006. 10.3390/ph3071988 27713338 PMC4036669

[57] Kamboj M Cohen N Gilhuley K Babady NE Seo SK Sepkowitz KA. Emergence of daptomycin-resistant VRE: experience of a single institution. Infect Control Hosp Epidemiol. (2011) 32:391. 10.1086/659152 21460492 PMC3676937

[58] Noviello S Ianniello F Leone S Fiore M Esposito S. *In vitro* activity of tigecycline: MICs, MBCs, time-kill curves and post-antibiotic effect. J Chemother. (2008) 20:577–80. 10.1179/joc.2008.20.5.577 19028619

[59] Fiore M Maraolo AE Gentile I Borgia G Leone S Sansone P. Current concepts and future strategies in the antimicrobial therapy of emerging Gram-positive spontaneous bacterial peritonitis. World J Hepatol. (2017) 9:1166. 10.4254/wjh.v9.i30.1166 29109849 PMC5666303

[60] Mercuro NJ Davis SL Zervos MJ Herc ES. Combatting resistant enterococcal infections: a pharmacotherapy review. Expert Opin Pharmacother. (2018) 19:979–92. 10.1080/14656566.2018.1479397 29877755

[61] Kessel J Bender J Werner G Griskaitis M Herrmann E Lehn A. Risk factors and outcomes associated with the carriage of tigecycline-and vancomycin-resistant *Enterococcus faecium*. J Infect. (2020) 82:227–34. 10.1016/j.jinf.2020.12.003 33285218

[62] Hoban DJ Reinert RR Bouchillon SK Dowzicky MJ. Global *in vitro* activity of tigecycline and comparator agents: tigecycline evaluation and surveillance trial 2004–2013. Ann Clin Microbiol Antimicrob. (2015) 14:1–16. 10.1186/s12941-015-0085-1 25958201 PMC4489028

[63] Shariati A Dadashi M Chegini Z van Belkum A Mirzaii M Khoramrooz SS. The global prevalence of Daptomycin, Tigecycline, Quinupristin/Dalfopristin, and Linezolid-resistant *Staphylococcus aureus* and coagulase–negative staphylococci strains: a systematic review and meta-analysis. Antimicrob Resist Infect Control. (2020) 9:1–20. 10.1186/s13756-020-00714-9 32321574 PMC7178749

