# Supplementary material for: The Global Prevalence of Daptomycin, Tigecycline, and Linezolid-Resistant Enterococcus faecalis and Enterococcus faecium Strains From Human Clinical Samples: A Systematic Review and Meta-Analysis
Source: Front Med (Lausanne). 2021 Sep 10;8:720647. doi: 10.3389/fmed.2021.720647 (PMC8460910; doi:10.3389/fmed.2021.720647)
Supplement: Supplementary file 1 [file Data_Sheet_1.docx]

**Table S1.** Characteristics of the articles that were included in the meta-analysis and reported resistance to tigecycline.

| **First name** | **Time of study** | **Country, continent** | **Total patient/sample** | **Total enterococcus** | ***E. faecalis*** | ***E. faecalis resistant*** | ***E. faecalis* intermediate resistant** | ***E. faecium*** | ***E. faecium* resistant** | ***E. faecium* intermediate resistant** | **Susceptibility testing method** | **Isolation source** | **Ref** |
| --- | --- | --- | --- | --- | --- | --- | --- | --- | --- | --- | --- | --- | --- |
| Hassan, 2018 | 2013-2015 | Egypt, Africa | 67 | 47 | 44 | 1 | 12 | NR | NR | 1 | BMD/DD/Multiplex-PCR/MALDI-TOF MS | Clinical isolates | (1) |
| Karlowsky, 2014 | 2011-2013 | Canada, America | 5209 | 304 | 304 | 2 | NR | NR | NR | NR | BMD/PCR | Clinical isolates | (2) |
| Barber, 2016 | 2014 | USA, America | 642 | 322 | 220 | NR | NR | 120 | 4 | NR | BMD/PCR | Clinical isolates | (3) |
| Sader, 2014 | 2006-2012 | USA, America | 68608 | 7185 | 4529 | 4 | 18 | 2656 | 5 | 13 | BMD/MALDI-TOF MS | Blood/urine/wound/skin | (4) |
| Zhao, 2012 | 2005-2010 | China, Asia | 5608 | 1160 | 548 | NR | NR | 593 | NR | 1 | Agar dilution | Urine/Blood/Pus/Bile | (5) |
| Wang, 2016 | 2012-2015 | China, Asia | 911 | 911 | NR | NR | NR | 911 | 9 | NR | BMD/DD/Vitek 2/PCR | Clinical isolates | (6) |
| Li, 2016 | 2015 | China, Asia | 2140 | 203 | 104 | 1 | NR | 99 | NR | NR | BMD/Agar dilution | Pneumonia/Skin/Soft tissue/Blood | (7) |
| Kuo, 2014 | 2012 | Taiwan, Asia | 134 | 134 | NR | NR | NR | 134 | 9 | NR | MIC/DD/E-test/PCR | Blood | (8) |
| Tsai, 2012 | 2006-2010 | Taiwan, Asia | 219 | 219 | NR | NR | NR | 219 | 3 | NR | BMD/DD | Clinical isolates | (9) |
| Yasemin, 2013 | 2007 | Turkey, asia | 60 | 60 | 14 | 5 | 7 | 46 | 1 | 5 | E-test | Blood/Rectal swab | (10) |
| Cattoir, 2014 | 2004-2012 | France, Europe | 17135 | 1301 | 969 | 2 | NR | 332 | NR | NR | BMD | Clinical isolates | (11) |
| Aznar, 2012 | 2004-2009 | Germany, Europe | 2961 | 576 | 351 | NR | NR | 225 | NR | 1 | BMD | Medical Centers | (12) |
| Maraki, 2014 | 2013 | Greek, Europe | 67 | 70 | 8 | NR | NR | 62 | 9 | NR | Vitek/E-test | Urine/skine/blood/decubitus ulcers/ascitic fluid/sputum/bile/CSF/soft tissue | (13) |
| Aznar, 2012 | 2004-2009 | Italy, Europe | 2961 | 739 | 492 | NR | 1 | 247 | 2 | 2 | BMD | Medical Centers | (12) |
| Stefani, 2013 | 2004-2011 | Italy, Europe | 13245 | 1076 | 740 | NR | NR | 336 | NR | 4 | BMD | Clinical isolates | (14) |
| Freitas, 2011 | 1996-2008 | Portugal, Europe | 890 | 890 | 405 | 6 | NR | NR | NR | NR | MIC/PCR | Clinical isolates | (15) |
| Tubau, 2010 | 2005-2006 | Spain, Europe | 700 | 92 | 53 | 1 | 10 | 39 | NR | 3 | E-test | Clinical isolates | (16) |
| Marco, 2016 | 2004-2014 | Spain, Europe | 23000 | 1740 | 1205 | 2 | 4 | 535 | NR | 2 | BMD | Medical Centers | (17) |
| Aznar, 2012 | 2004-2009 | Spain, Europe | 2961 | 694 | 514 | NR | 2 | 180 | NR | 1 | BMD | Medical Centers | (12) |
| Monticelli, 2020 | 2011-2017 | Italy, Europe | 4858 | 4858 | 2866 | 2 | NR | 1739 | 16 | NR | MIC | Blood | (18) |

Abbreviations: DD; disk diffusion, BMD; broth microdilution,

**Table S2**: Characteristics of the articles that were included in the meta-analysis and reported resistance to linezolid.

| **First name** | **Time of study** | **Country, continent** | **Total patient/sample** | **Total enterococcus** | ***E. faecalis*** | ***E. faecalis resistant*** | ***E. faecalis intermediatte***  ***resistant*** | ***E. faecium*** | ***E. faecium* resistant** | ***E. faecium* intermediate**  **resistant** | **Susceptibility testing method** | **Isolation source** | **Ref** |
| --- | --- | --- | --- | --- | --- | --- | --- | --- | --- | --- | --- | --- | --- |
| Arias, 2012 | 2000-2010 | USA, America | 7424 | 884 | 516 | 296 | 171 | 368 | 2 | 3 | BMD | Clinical isolates | (19) |
| Jevitt, 2003 | 1996-2001 | USA, America | 178 | 90 | 63 | 3 | NR | NR | NR | NR | BMD | Clinical isolates | (20) |
| Kainer, 2007 | 2004-2005 | USA, America | 406 | 406 | NR | 13 | NR | NR | 2 | NR | MIC | Blood/Rectal swab | (21) |
| Draghi, 2005 | 2004 | USA, America | 3802 | 624 | 248 | 1 | 1 | 196 | 4 | 3 | BMD | Blood/urine/wound/respiratory fluid | (22) |
| Doern, 2016 | 2012 | USA, America | 27 | 11 | 5 | 1 | 1 | 6 | 5 | NR | Vitek/E-test/DD/BMD/PCR | Blood/urine/abscess/Biopsy specimen/Thracetes fluid | (23) |
| Farrell, 2009 | 2008 | USA, America | 6113 | 901 | 586 | NR | NR | 270 | 6 | NR | BMD/DD/E-test | Medical Centers | (24) |
| Deshpande, 2007 | 2003 | USA, America | 839 | 832 | 56 | 1 | NR | 776 | 6 | NR | BMD/E-test/PCR | Urinary tract/Skin/Soft tissue | (25) |
| Barber, 2016 | 2014 | USA, America | 642 | 322 | 220 | NR | NR | 120 | 10 | NR | BMD/PCR | Clinical isolates | (3) |
| Mendes, 2014 | 2012 | USA, America | 7429 | 937 | 640 | 1 | NR | 259 | 4 | NR | BMD/Vitek/MALDI-TOF MS | Clinical isolates | (26) |
| Mendes, 2016 | 2014-2015 | USA, America | 150 | 45 | 13 | 7 | NR | 32 | 24 | NR | MIC/Vitek/ MALDI-TOF MS | Clinical isolates | (27) |
| Mendes, 2012 | 2007-2009 | USA, America | 10705 | 2308 | 1442 | NR | NR | 866 | 15 | NR | BMD/Vitek | Clinical isolates | (28) |
| Edelsberg, 2014 | 2007-2010 | USA, America | 96788 | 11281 | 7007 | 126 | NR | 4274 | 47 | NR | MIC | Clinical isolates | (29) |
| Zhanel, 2003 | 2002 | USA, America | 860 | 860 | 202 | NR | NR | 658 | 2 | 2 | BMD/Multiplex PCR | Urine | (30) |
| Wang, 2018 | 2013 | USA, America | 250 | 250 | NR | NR | NR | 92 | 1 | NR | BMD/E-test/MALDI Biotyper CA | Blood/Wound/Urine/Pelvic/Peritoneal fluid | (31) |
| Sader, 2008 | 2002-2006 | USA, America | 11906 | 2722 | 1871 | 4 | NR | 851 | 10 | NR | BMD | Blood | (32) |
| Hayakawa, 2012 | 2008-2009 | USA, America | 40 | 40 | NR | 8 | NR | NR | 12 | NR | BMD | Clinical isolates | (33) |
| Sader, 2009 | 2007-2008 | USA, America | 12443 | 4551 | 1401 | NR | NR | 843 | 14 | 1 | BMD | Medical Centers | (34) |
| Sader, 2011 | 2005-2010 | USA, America | 29632 | 6774 | 4312 | 1 | NR | 2462 | 34 | 7 | BMD | Medical Centers | (35) |
| Sader, 2014 | 2006-2012 | USA, America | 68608 | 7185 | 4529 | 4 | NR | 2656 | 10 | NR | BMD/MALDI-TOF MS | Blood/urine/wound/skin | (4) |
| Critchley, 2003 | 2000-2001 | USA, America | 6973 | 2540 | 2092 | NR | NR | 368 | 1 | NR | BMD/Vitek/PCR | Blood/Wound/Respiratory tract/Urine/Cerebrospinal | (36) |
| Dipippo, 2016 | 2013-2015 | USA, America | 77 | 77 | NR | NR | NR | 77 | 13 | NR | E-test | Clinical isolates | (37) |
| Dipippo, 2017 | 2013-2015 | USA, America | 272 | 139 | 95 | NR | NR | 44 | 13 | NR | E-test/Vitek/BMD | Bood/urine | (38) |
| Dowzicky, 2008 | 2005-2007 | USA, America | 29846 | 2628 | 1903 | 4 | 18 | 725 | 1 | 23 | BMD/DD | Clinical isolates | (39) |
| Petersen, 2010 | 2007-2009 | USA, America | 213 | 213 | 196 | 4 | NR | NR | NR | NR | DD/PCR | Clinical isolates | (40) |
| Descourouez, 2013 | 2007-2010 | USA, America | 32 | 32 | NR | NR | NR | 32 | 1 | NR | BMD | Urinary stent | (41) |
| Flamm, 2012 | 2010 | USA, America | 6801 | 934 | NR | 2 | NR | NR | 10 | NR | BMD/E-test | Clinical isolates | (42) |
| Waites, 2006 | 2004-2005 | USA, America | 11215 | 1020 | 740 | 4 | 14 | 280 | NR | 10 | BMD | Medical Centers | (43) |
| Kamboj, 2011 | 2007-2009 | USA, America | 18 | 18 | NR | NR | NR | 18 | 1 | NR | BMD | Clinical isolates | (44) |
| Marini, 2016 | 2012-2015 | USA, America | 98 | 98 | NR | NR | NR | 98 | 1 | NR | Microscan/E-test | Blood | (45) |
| Mutnick, 2003 | 2000-2001 | USA, America | 9833 | 1277 | NR | 2 | NR | NR | 4 | NR | E-test/DD/BMD | Blood/skin/wound/soft tissue/respiratory | (46) |
| Jones, 2001 | 2000 | USA, America | 3100 | 501 | 332 | 1 | 17 | 169 | 2 | 9 | DD | Clinical isolates | (47) |
| Jones, 2007 | 2006 | USA, America | 5374 | 808 | NR | 3 | NR | NR | 10 | NR | E-test/DD | Clinical isolates | (48) |
| Pai, 2002 | 2000-2001 | USA, America | 34 | 34 | NR | NR | NR | 34 | 4 | NR | Agar dilution | Clinical isolates | (49) |
| Smith, 2005 | 1999-2003 | USA, America | 258 | 116 | 24 | 1 | NR | 52 | 1 | NR | BMD | Blood | (50) |
| Flamm, 2016 | 2014 | USA, America | 6865 | 855 | 589 | 1 | NR | 239 | 5 | NR | BMD/E-test/PCR | Medical Centers | (51) |
| Mendes, 2015 | 2013 | USA, America | 7967 | 757 | 434 | NR | NR | 299 | 1 | NR | BMD | Clinical isolates | (52) |
| Richter, 2003 | 1996-2000 | USA, America | 1483 | 984 | 540 | NR | 3 | 480 | NR | 27 | BMD | Clinical isolates | (53) |
| Dilworth, 2019 | 2017-2018 | USA, America | 86 | 103 | NR | NR | NR | 81 | 11 | NR | E-test/DD/PCR | Rectal swabs | (54) |
| Li, 2018 | NR | China, Asia | 1449 | 99 | 86 | 3 | NR | 13 | NR | NR | DD/Vitek | Burn Patients | (55) |
| Bai, 2018 | 2011-2016 | China, Asia | 357 | 357 | 289 | 57 | NR | 68 | 3 | NR | BMD/Vitek/PCR | Clinical isolates | (56) |
| Zhao, 2012 | 2005-2010 | China, Asia | 5608 | 1160 | 548 | NR | 9 | 593 | NR | 5 | Agar dilution | Urine/Blood/Pus/Bile | (5) |
| Zhang, 2019 | 2015-2016 | China, Asia | 1758 | 183 | 74 | 1 | 4 | 109 | 1 | 1 | BMD/Agar dilution | Blood | (57) |
| Chen, 2019 | 2009-2013 | China, Asia | 1067 | 16 | 13 | 11 | NR | 3 | 2 | NR | Vitek | Clinical isolates | (58) |
| Cai, 2019 | 2015 | China, Asia | 3458 | 122 | 65 | 38 | NR | 26 | 11 | NR | BMD/PCR/WGS | Stool | (59) |
| Zhao, 2019 | 2012-2017 | China, Asia | 709 | 110 | 35 | 1 | NR | 44 | 1 | NR | VITEK-2 | Bile | (60) |
| Zheng, 2017 | 2010-2016 | China, Asia | 265 | 265 | 265 | 10 | NR | NR | NR | NR | Vitek/PCR/RT-PCR | Clinical isolates | (61) |
| Shi, 2017 | 2014 | China, Asia | 575 | 70 | 52 | 1 | NR | 18 | 3 | NR | MIC/Vitek | Clinical isolates | (62) |
| Huang, 2019 | 2015-2017 | China, Asia | NR | 59810 | 31709 | 932 | NR | 28101 | 252 | NR | DD/Vitek-2/BD phoenix/ MALDI-TOF MS | Clinical isolates | (63) |
| Kang, 2014 | NR | China, Asia | 135 | 135 | NR | NR | NR | 135 | 1 | NR | MIC/Vitek | Clinical isolates | (64) |
| Zhu, 2018 | 2011-2017 | China, Asia | 4275 | 426 | 132 | 5 | NR | 294 | 9 | NR | DD/Vitek-2 | Blood | (65) |
| Wang, 2015 | 2010-2013 | China, Asia | 517 | 517 | 252 | 65 | NR | 265 | 53 | NR | BMD/Phoniex | Urine | (66) |
| Hua, 2019 | 2014-2017 | China, Asia | 1120 | 1120 | 1120 | 43 | NR | NR | NR | NR | MIC/Vitek/BMD/PCR | Clinical isolates | (67) |
| Wang, 2016 | 2012-2015 | China, Asia | 911 | 911 | NR | NR | NR | 911 | 8 | NR | BMD/DD/Vitek 2/PCR | Clinical isolates | (6) |
| Li, 2016 | 2015 | China, Asia | 2140 | 203 | 104 | 1 | 1 | 99 | 1 | 1 | BMD/Agar dilution | Pneumonia/Skin/Soft tissue/Blood | (7) |
| SU Mao-sheng, 2012 | 2008-2011 | China, Asia | 594 | 77 | 17 | 1 | NR | 60 | 2 | NR | DD | Peritoneal/Sputum/Bile/Wound/Urine/Blood/Abscess/Pleural/Tissue/Catheter | (68) |
| Qu, 2006 | 2002-2003 | China, Asia | 106 | 106 | 48 | NR | NR | 46 | NR | 2 | DD/Agar dilution/PCR | Abdominal fluid/Bile/Urine/Blood/Sputum/Secretion | (69) |
| Zhou, 2019 | 2014-2017 | China, Asia | 2555 | 2555 | NR | 24 | NR | NR | 3 | NR | E-tst/Vitek/PCR/WGS | Blood/bile/secretions/ascites/urine/drainage | (70) |
| Li, 2015 | 2013-2014 | China, Asia | 160 | 160 | 35 | NR | NR | 105 | 3 | NR | DD/Vitek-2/PCR | Rectal swab/Stool | (71) |
| Jia, 2015 | 2011-2014 | China, Asia | 17193 | 2151 | NR | 39 | NR | 1120 | 5 | NR | BMD/Vitek | Clinical isolates | (72) |
| Zhang, 2018 | 2011-2016 | China, Asia | 1623 | 1623 | 789 | 31 | NR | 834 | 2 | NR | BMD/PCR/ MALDI-TOF MS | Wound/Urine/Pus | (73) |
| Tian, 2014 | 2009-2013 | China, Asia | 1713 | 1689 | 812 | NR | 14 | 877 | NR | 1 | MIC | Blood | (74) |
| Phukan, 2016 | 2013 | India, Asia | 67 | 67 | 54 | 2 | NR | 13 | 1 | NR | DD/E-test/Agar dilution/PCR | Urine/Blood/Sputum/Pus/Throat swabs | (75) |
| Bhatt, 2015 | 2011-2014 | India, Asia | 200 | 200 | 150 | 1 | NR | 50 | 3 | NR | DD/E-test | Urine/Pus/Blood/CSF/Tracheal aspirate/Semen/Bile | (76) |
| Yadav, 2017 | 2014-2015 | India, Asia | 15342 | 200 | 169 | 4 | NR | 27 | NR | NR | BMD/Vitek | Blood/urine/pus/genital swab | (77) |
| Jahansepas, 2018 | 2014-2015 | Iran, Asia | 160 | 160 | 11 | 4 | NR | 19 | 11 | NR | MIC/E-test/Multiplex-PCR | Clinical isolates | (78) |
| Esmailzadeh, 2016 | 2013-2014 | Iran, Asia | 180 | 180 | NR | 6 | NR | NR | 1 | NR | DD/PCR | Rectal Swab | (79) |
| Hosseini Doust, 2007 | NR | Iran, Asia | 195 | 195 | NR | NR | NR | NR | 1 | NR | DD/PCR | Clinical isolates | (80) |
| Yasliani, 2009 | 2006-2007 | Iran, Asia | 200 | 200 | NR | NR | NR | 22 | 2 | NR | DD/Agar Dilution/PCR | Blood/urine/stool/wound | (81) |
| Coskun, 2019 | 2016-2017 | Turkey, Asia | 93 | 93 | 31 | 2 | NR | 62 | 43 | NR | MIC/PCR | Blood/urine/wound/respiratory fluid | (82) |
| Cakirlar, 2016 | 2014-2015 | Turkey, Asia | 97 | 97 | 53 | 1 | NR | NR | NR | NR | Phoniex/MIC | Blood | (83) |
| Sibel, 2012 | 2008-2010 | Turkey, Asia | 118 | 118 | 88 | 9 | NR | 22 | 2 | N | Vitek | Clinical isolates | (84) |
| Verma, 2007 | 2002-2005 | England, Europe | 1(CASE with over a 3-year period) | 72 | 31 | 31 | NR | 41 | 41 | NR | E-test/Vitek | Fecal samples/throat/rectal swab/groin swab | (85) |
| Decousser, 2018 | 2004-2016 | France, Europe | 26486 | 2545 | 1802 | 4 | NR | 696 | 2 | NR | BMD | Clinical isolates | (86) |
| Sassi, 2019 | 2006-2016 | France, Europe | 3974 | 3974 | NR | 3 | NR | NR | 6 | NR | BMD/MALDI-TOF MS/PCR | Dialysis catheter/Rectal swab/Urine | (87) |
| Sader, 2006 | 2002-2004 | France, Europe | 9322 | 247 | NR | 1 | NR | NR | NR | NR | BMD | Skin/Blood | (88) |
| Schulte, 2008 | 2004-2006 | Germany, Europe | 18 | 18 | 1 | 1 | NR | 17 | 17 | NR | Vitek/E-test/BMD/agar diffusion | Blood/urine/stool/Intra-abdominal swab/Tracheal fluid | (89) |
| Bodmann, 2012 | 2006-2010 | Germany, Europe | 1028 | 125 | 125 | 1 | NR | NR | NR | NR | MIC | Clinical isolates | (90) |
| Theilacker, 2009 | 2004-2005 | Germany, Europe | 25 | 25 | NR | NR | NR | 25 | 4 | NR | MIC | Clinical isolates | (91) |
| Werner, 2007 | NR | Germany, Europe | 83 | 83 | NR | NR | NR | NR | 3 | NR | BMD/PCR | Blood | (92) |
| Brauers, 2005 | 2001-2002 | Germany, Europe | 8594 | 8594 | 1188 | 25 | 27 | 524 | 9 | 16 | BMD/Agar Diffusion | Blood/urine/wound/respiratory fluid | (93) |
| Aznar, 2012 | 2004-2009 | Germany, Europe | 2961 | 576 | 351 | 1 | 1 | 225 | NR | NR | BMD | Medical Centers | (12) |
| Klare, 2015 | 2008-2014 | Germany, Europe | 4461 | 4461 | 4461 | 251 | NR | NR | NR | NR | E-test/BMD/PCR | Stool/urine/rectal swab | (94) |
| Kresken, 2009 | 2005 | Germany, Europe | 2610 | 295 | 150 | NR | NR | 145 | 1 | NR | BMD | Blood/Wound swab | (95) |
| Aznar, 2012 | 2004-2009 | Italy, Europe | 2961 | 739 | 492 | NR | NR | 247 | 2 | NR | BMD | Medical Centers | (12) |
| Campanile, 2019 | NR | Italy, Europe | 46 | 13 | 7 | 1 | NR | 6 | 1 | NR | BMD | Clinical isolates | (96) |
| Manfredi, 2004 | 2000-2001 | Italy, Europe | 5594 | 5524 | 4846 | 62 | NR | 678 | 24 | NR | Vitek-2 | Clinical isolates | (97) |
| Rodloff, 2008 | 2004-2006 | Italy, Europe | 5594 | 154 | 105 | NR | 3 | 49 | NR | 4 | BMD | Blood/Respiratory tract/Urine/Skin/Wound/Fluids | (98) |
| Borbone, 2008 | 2004-2005 | Italy, Europe | 117 | 30 | 15 | NR | 1 | 15 | NR | 1 | BMD/PCR | Skin/Blood/Intra-abdominal fluid/Respiratory | (99) |
| Stefani, 2013 | 2004-2011 | Italy, Europe | 13245 | 1076 | 740 | 0 | NR | 336 | 2 | 1 | BMD | Clinical isolates | (14) |
| Cercenado, 2007 | 2005-2006 | Spain, Europe | 8064 | 1850 | 1439 | 3 | NR | 340 | NR | NR | BMD/E-test/Real -Time PCR | Clinical isolates | (100) |
| Marco, 2016 | 2004-2014 | Spain, Europe | 23000 | 1740 | 1205 | 1 | NR | 535 | 3 | NR | BMD | Medical Centers | (17) |
| Aznar, 2012 | 2004-2009 | Spain, Europe | 2961 | 694 | 514 | 1 | NR | 180 | 1 | 1 | BMD | Medical Centers | (12) |
| Johnson, 2004 | NR | UK, Europe | 545 | 89 | 46 | 9 | NR | 43 | 6 | NR | MIC | Clinical isolates | (101) |
| Rodloff, 2008 | 2004-2006 | UK, Europe | 5594 | 53 | 37 | NR | NR | 16 | NR | 1 | BMD | Blood/Respiratory tract/Urine/Skin/Wound/Fluids | (98) |
| Bassetti, 2003 | 2000-2001 | USA, America | 14 | 14 | NR | NR | NR | 14 | 2 | NR | E-test/ agar-dilution | peri-rectal | (102) |
| Sami, 2020 | NR | India, Asia | 1014 | 1014 | NR | NR | NR | NR | 8 | NR | DD/ Vitek 2/PCR | Clinical isolates | (103) |
| Knight, 2020 | 2006-2017 | USA, America | 697 | 42 | 16 | NR | NR | 21 | 8 | NR | MIC | Blood | (104) |
| Zou, 2020 | 2014-2018 | China, Asia | 1902 | 1902 | 1902 | 91 | NR | NR | NR | NR | Vitek 2/PCR | clinical isolates | (105) |
| Rana, 2020 | 2019 | India, Asia | 100 | 100 | 70 | 2 | NR | 21 | 1 | NR | DD | Clinical isolates | (106) |
| Monticelli, 2020 | 2011-2017 | Italy, Europe | 4858 | 4858 | 2866 | 26 | NR | 1739 | 3 | NR | MIC | Blood | (18) |
| Li, 2020 | 2017 | China, Asia | 573 | 573 | 296 | 2 | 6 | 264 | NR | NR | BMD/ Vitek-2/PCR | Clinical isolates | (107) |

Abbreviations: DD; disk diffusion, BMD; broth microdilution, WGS; whole genome sequencing

**Table S3:** Characteristics of the articles that were included in the meta-analysis and reported resistance to daptomycin.

| **First name** | **Time of study** | **Country, continent** | **Total patient/sample** | **Total enterococcus** | ***E. faecalis*** | **E. faecalis resistant** | ***E. faecalis* intermediate resistant** | ***E. faecium*** | ***E. faecium* resistant** | ***E. faecium* intermediate**  **resistant** | **Susceptibility testing method** | **Isolation source** | **Ref** |
| --- | --- | --- | --- | --- | --- | --- | --- | --- | --- | --- | --- | --- | --- |
| Arias, 2012 | 2000-2010 | USA, America | 7424 | 884 | 516 | NR | NR | 368 | NR | 1 | BMD | Clinical isolates | (19) |
| Barber, 2016 | 2014 | USA, America | 642 | 322 | 220 | NR | NR | 120 | 25 | NR | BMD/PCR | Clinical isolates | (3) |
| Edelsberg, 2014 | 2007-2010 | USA, America | 96788 | 11281 | 7007 | 14 | NR | 4274 | 166 | NR | MIC | Clinical isolates | (29) |
| Wang, 2018 | 2013 | USA, America | 250 | 250 | NR | NR | NR | 92 | 26 | NR | BMD/E-test/MALDI Biotyper CA | Blood/Wound/Urine/Pelvic/Peritoneal fluid | (31) |
| Sader, 2008 | 2002-2006 | USA, America | 11906 | 2722 | 1871 | 1 | NR | 851 | 5 | NR | BMD | Blood | (32) |
| Sader, 2011 | 2005-2010 | USA, America | 29632 | 6774 | 4312 | NR | NR | 2462 | NR | 4 | BMD | Medical Centers | (35) |
| Dipippo, 2016 | 2013-2015 | USA, America | 77 | 77 | NR | NR | NR | 77 | 17 | NR | E-test | Clinical isolates | (37) |
| Dipippo, 2017 | 2013-2015 | USA, America | 272 | 139 | 95 | 16 | NR | 44 | 13 | NR | E-test/Vitek/BMD | Bood/urine | (38) |
| Lewis, 2019 | 2010-2015 | USA, America | 372 | 46 | NR | NR | NR | 46 | 14 | NR | MIC | Blood/Abdominal fluid/Wound Swab/Urine | (108) |
| Rolston, 2013 | 2011 | USA, America | 1082 | 131 | 12 | NR | NR | 28 | 1 | NR | E-test | Blood | (109) |
| Patel, 2016 | 2011-2013 | USA, America | 65 | 65 | 6 | NR | NR | 59 | 1 | NR | MIC | Blood | (110) |
| Marini, 2016 | 2012-2015 | USA, America | 98 | 98 | NR | NR | NR | 98 | 22 | NR | Microscan/E-test | Blood | (45) |
| Sader, 2015 | 2009-2013 | USA, America | 46467 | 4385 | 2848 | 1 | NR | 1537 | 5 | NR | BMD/Vitek 2/ MALDI-TOF MS | Medical Centers | (111) |
| Kelesidis, 2013 | NR | USA, America | 11 | 11 | 5 | 5 | NR | 6 | 6 | NR | E-tset | Urinary tract | (112) |
| Kelesidis, 2012 | 2007-2011 | USA, America | 3600 | 3600 | NR | 2 | NR | NR | 6 | NR | BMD | Blood/Urine/Wound | (113) |
| Jahansepas, 2018 | 2014-2015 | Iran, Asia | 160 | 160 | 11 | 4 | NR | 19 | 16 | NR | MIC/E-test/Multiplex-PCR | Clinical isolates | (78) |
| Udaondo, 2020 | 2018 | USA, America | 2 | 20 | NR | NR | NR | 20 | 2 | NR | E-tests/ BacT-ALERT 3D/ Vitek 2/ Vitek 2 MS/ WGS | Blood | (114) |

Abbreviations: DD; disk diffusion, BMD; broth microdilution, WGS; whole genome sequencing

1. Hassan RM, Ghaith DM, Ismail DK, Zafer MM. Reduced susceptibility of Enterococcus spp. isolates from Cairo University Hospital to tigecycline: Highlight on the influence of proton pump inhibitors. Journal of global antimicrobial resistance. 2018;12:68-72.

2. Karlowsky J, Walkty A, Baxter M, Adam H, Zhanel G. In vitro activity of oritavancin against Gram-positive pathogens isolated in Canadian hospital laboratories from 2011 to 2013. Diagnostic microbiology and infectious disease. 2014;80(4):311-5.

3. Barber KE, Smith JR, Raut A, Rybak MJ. Evaluation of tedizolid against Staphylococcus aureus and enterococci with reduced susceptibility to vancomycin, daptomycin or linezolid. Journal of Antimicrobial Chemotherapy. 2016;71(1):152-5.

4. Sader HS, Farrell DJ, Flamm RK, Jones RN. Variation in potency and spectrum of tigecycline activity against bacterial strains from US medical centers since its approval for clinical use (2006 to 2012). Antimicrobial agents and chemotherapy. 2014;58(4):2274-80.

5. Zhao C, Sun H, Wang H, Liu Y, Hu B, Yu Y, et al. Antimicrobial resistance trends among 5608 clinical Gram-positive isolates in China: results from the Gram-Positive Cocci Resistance Surveillance program (2005-2010). Diagnostic microbiology and infectious disease. 2012;73(2):174-81.

6. Wang S, Guo Y, Lv J, Qi X, Li D, Chen Z, et al. Characteristic of Enterococcus faecium clinical isolates with quinupristin/dalfopristin resistance in China. BMC microbiology. 2016;16(1):246.

7. Li S, Guo Y, Zhao C, Chen H, Hu B, Chu Y, et al. In vitro activities of tedizolid compared with other antibiotics against Gram-positive pathogens associated with hospital-acquired pneumonia, skin and soft tissue infection and bloodstream infection collected from 26 hospitals in China. Journal of medical microbiology. 2016;65(10):1215-24.

8. Kuo A-J, Su L-H, Shu J-C, Wang J-T, Wang J-H, Fung C-P, et al. National surveillance on vancomycin-resistant Enterococcus faecium in Taiwan: emergence and widespread of ST414 and a Tn1546-like element with simultaneous insertion of IS1251-like and IS1678. PloS one. 2014;9(12).

9. Tsai H-Y, Liao C-H, Chen Y-H, Lu P-L, Huang C-H, Lu C-T, et al. Trends in susceptibility of vancomycin-resistant Enterococcus faecium to tigecycline, daptomycin, and linezolid and molecular epidemiology of the isolates: results from the Tigecycline In Vitro Surveillance in Taiwan (TIST) study, 2006 to 2010. Antimicrobial agents and chemotherapy. 2012;56(6):3402-5.

10. Yasemin C, Serap G, Oznur A, Yakup C, Serdar O. Investigation of Linezolid, Daptomycin, Quinupristin-Dalfopristin and Tigecycline Susceptibilities against Vancomycin Resistant Enterococcus Isolates. JOURNAL OF PURE AND APPLIED MICROBIOLOGY. 2013;7(2):1367-71.

11. Cattoir V, Dowzicky MJ. A longitudinal assessment of antimicrobial susceptibility among important pathogens collected as part of the Tigecycline Evaluation and Surveillance Trial (TEST) in France between 2004 and 2012. Antimicrobial resistance and infection control. 2014;3(1):36.

12. Aznar J, Lepe JA, Dowzicky MJ. Antimicrobial susceptibility among E. faecalis and E. faecium from France, Germany, Italy, Spain and the UK (TEST Surveillance Study, 2004-2009). Journal of Chemotherapy. 2012;24(2):74-80.

13. Maraki S, Samonis G, Dimopoulou D, Mantadakis E. Susceptibility of glycopeptide-resistant enterococci to linezolid, quinupristin/dalfopristin, tigecycline and daptomycin in a tertiary Greek hospital. Infection & chemotherapy. 2014;46(4):253-6.

14. Stefani S, Dowzicky MJ. Longitudinal assessment of antimicrobial susceptibility among Gram-negative and Gram-positive organisms collected from Italy as part of the tigecycline evaluation and surveillance trial between 2004 and 2011. Pharmaceuticals. 2013;6(11):1381-406.

15. Freitas A. Development of resistance in Enterococcus faecium infection: case report. Reactions. 2016;1612:119-30.

16. Tubau F, Liñares J, Rodríguez M-D, Cercenado E, Aldea M-J, González-Romo F, et al. Susceptibility to tigecycline of isolates from samples collected in hospitalized patients with secondary peritonitis undergoing surgery. Diagnostic microbiology and infectious disease. 2010;66(3):308-13.

17. Marco F, Dowzicky MJ. Antimicrobial susceptibility among important pathogens collected as part of the Tigecycline Evaluation and Surveillance Trial (TEST) in Spain, 2004–2014. Journal of global antimicrobial resistance. 2016;6:50-6.

18. Monticelli J, Di Bella S, Giacobbe DR, Amato G, Antonello RM, Barone E, et al. Trends in the Incidence and Antibiotic Resistance of Enterococcal Bloodstream Isolates: A 7-Year Retrospective Multicenter Epidemiological Study in Italy. Microbial Drug Resistance. 2020.

19. Arias CA, Mendes RE, Stilwell MG, Jones RN, Murray BE. Unmet needs and prospects for oritavancin in the management of vancomycin-resistant enterococcal infections. Clinical infectious diseases. 2012;54(suppl_3):S233-S8.

20. Jevitt LA, Smith AJ, Williams PP, Raney PM, McGowan Jr JE, Tenover FC. In vitro activities of daptomycin, linezolid, and quinupristin-dalfopristin against a challenge panel of staphylococci and enterococci, including vancomycin-intermediate Staphylococcus aureus and vancomycin-resistant Enterococcus faecium. Microbial Drug Resistance. 2003;9(4):389-93.

21. Kainer MA, Devasia RA, Jones TF, Simmons BP, Melton K, Chow S, et al. Response to emerging infection leading to outbreak of linezolid-resistant enterococci. Emerging infectious diseases. 2007;13(7):1024.

22. Draghi DC, Sheehan DJ, Hogan P, Sahm DF. In vitro activity of linezolid against key gram-positive organisms isolated in the United States: results of the LEADER 2004 surveillance program. Antimicrobial agents and chemotherapy. 2005;49(12):5024-32.

23. Doern CD, Park JY, Gallegos M, Alspaugh D, Burnham C-AD. Investigation of linezolid resistance in staphylococci and enterococci. Journal of clinical microbiology. 2016;54(5):1289-94.

24. Farrell DJ, Mendes RE, Ross JE, Jones RN. Linezolid surveillance program results for 2008 (LEADER Program for 2008). Diagnostic microbiology and infectious disease. 2009;65(4):392-403.

25. Deshpande LM, Fritsche TR, Moet GJ, Biedenbach DJ, Jones RN. Antimicrobial resistance and molecular epidemiology of vancomycin-resistant enterococci from North America and Europe: a report from the SENTRY antimicrobial surveillance program. Diagnostic microbiology and infectious disease. 2007;58(2):163-70.

26. Mendes RE, Flamm RK, Hogan PA, Ross JE, Jones RN. Summary of linezolid activity and resistance mechanisms detected during the 2012 LEADER surveillance program for the United States. Antimicrobial agents and chemotherapy. 2014;58(2):1243-7.

27. Mendes RE, Farrell DJ, Sader HS, Flamm RK, Jones RN. Oritavancin Activity Tested against Molecularly Characterized Staphylococci and Enterococci Displaying Elevated Linezolid MIC Results. Antimicrob Agents Chemother. 2016;60(6):3817-20.

28. Mendes RE, Sader HS, Farrell DJ, Jones RN. Telavancin activity tested against a contemporary collection of Gram-positive pathogens from USA hospitals (2007–2009). Diagnostic microbiology and infectious disease. 2012;72(1):113-7.

29. Edelsberg J, Weycker D, Barron R, Li X, Wu H, Oster G, et al. Prevalence of antibiotic resistance in US hospitals. Diagnostic microbiology and infectious disease. 2014;78(3):255-62.

30. Zhanel GG, Laing NM, Nichol KA, Palatnick LP, Noreddin A, Hisanaga T, et al. Antibiotic activity against urinary tract infection (UTI) isolates of vancomycin-resistant enterococci (VRE): results from the 2002 N orth A merican V ancomycin R esistant E nterococci S usceptibility S tudy (NAVRESS). Journal of Antimicrobial Chemotherapy. 2003;52(3):382-8.

31. Wang G, Yu F, Lin H, Murugesan K, Huang W, Hoss AG, et al. Evolution and mutations predisposing to daptomycin resistance in vancomycin-resistant Enterococcus faecium ST736 strains. PloS one. 2018;13(12).

32. Sader HS, Fritsche T, Jones R. Frequency of Occurrence and Daptomycin Susceptibility Rates of Gram-positive Organisms Causing Bloodstream. Journal of Chemotherapy. 2008;20(5):570-6.

33. Hayakawa K, Marchaim D, Pogue JM, Ho K, Parveen S, Nanjireddy P, et al. Predictors and outcomes of linezolid-resistant vancomycin-resistant Enterococcus: a case-case-control study. American journal of infection control. 2012;40(10):e261-e3.

34. Sader HS, Jones RN. Antimicrobial susceptibility of Gram-positive bacteria isolated from US medical centers: results of the Daptomycin Surveillance Program (2007–2008). Diagnostic microbiology and infectious disease. 2009;65(2):158-62.

35. Sader HS, Moet GJ, Farrell DJ, Jones RN. Antimicrobial susceptibility of daptomycin and comparator agents tested against methicillin-resistant Staphylococcus aureus and vancomycin-resistant enterococci: trend analysis of a 6-year period in US medical centers (2005–2010). Diagnostic microbiology and infectious disease. 2011;70(3):412-6.

36. Critchley IA, Blosser-Middleton RS, Jones ME, Thornsberry C, Sahm DF, Karlowsky JA. Baseline study to determine in vitro activities of daptomycin against gram-positive pathogens isolated in the United States in 2000-2001. Antimicrobial agents and chemotherapy. 2003;47(5):1689-93.

37. Dipippo AJ, Tverdek FP, Tarrand JJ, Munita JM, Arias C, Shelburne SA, et al., editors. Previous Daptomycin Exposure Predicts Daptomycin Non-Susceptible Enterococcus faecium Bloodstream Infections in Adult Leukemia Patients. Open Forum Infectious Diseases; 2016: Oxford University Press.

38. DiPippo AJ, Tverdek FP, Tarrand JJ, Munita JM, Tran TT, Arias CA, et al. Daptomycin non-susceptible Enterococcus faecium in leukemia patients: Role of prior daptomycin exposure. Journal of Infection. 2017;74(3):243-7.

39. Dowzicky MJ, Park CH. Update on antimicrobial susceptibility rates among gram-negative and gram-positive organisms in the United States: results from the Tigecycline Evaluation and Surveillance Trial (TEST) 2005 to 2007. Clinical therapeutics. 2008;30(11):2040-50.

40. Petersen PJ, Ruzin A, Tuckman M, Jones CH. In vitro activity of tigecycline against patient isolates collected during phase 3 clinical trials for diabetic foot infections. Diagnostic microbiology and infectious disease. 2010;66(4):407-18.

41. Descourouez JL, Jorgenson MR, Wergin JE, Rose WE. Fosfomycin synergy in vitro with amoxicillin, daptomycin, and linezolid against vancomycin-resistant Enterococcus faecium from renal transplant patients with infected urinary stents. Antimicrobial agents and chemotherapy. 2013;57(3):1518-20.

42. Flamm RK, Farrell DJ, Mendes RE, Ross JE, Sader HS, Jones RN. LEADER surveillance program results for 2010: an activity and spectrum analysis of linezolid using 6801 clinical isolates from the United States (61 medical centers). Diagnostic microbiology and infectious disease. 2012;74(1):54-61.

43. Waites KB, Duffy LB, Dowzicky MJ. Antimicrobial susceptibility among pathogens collected from hospitalized patients in the United States and in vitro activity of tigecycline, a new glycylcycline antimicrobial. Antimicrobial agents and chemotherapy. 2006;50(10):3479-84.

44. Kamboj M, Cohen N, Gilhuley K, Babady NE, Seo SK, Sepkowitz KA. Emergence of daptomycin-resistant VRE: experience of a single institution. Infection Control & Hospital Epidemiology. 2011;32(4):391-4.

45. Marini RV, Shields RK, Clarke L, Clancy CJ, Nguyen M-H, Viehman JA. Clinical Outcomes of Bloodstream Infections Due to Vancomycin-Resistant Enterococcus faecium. Open Forum Infectious Diseases. 2016;3(suppl_1).

46. Mutnick AH, Enne V, Jones RN. Linezolid resistance since 2001: SENTRY antimicrobial surveillance program. Annals of Pharmacotherapy. 2003;37(6):769-74.

47. Jones RN, Ballow CH, Biedenbach DJ, Hospital ECM, Hospital SM, Hospital FC, et al. Multi-laboratory assessment of the linezolid spectrum of activity using the Kirby-Bauer disk diffusion method: Report of the Zyvox® Antimicrobial Potency Study (ZAPS) in the United States. Diagnostic microbiology and infectious disease. 2001;40(1-2):59-66.

48. Jones RN, Fritsche TR, Sader HS, Ross JE. LEADER surveillance program results for 2006: an activity and spectrum analysis of linezolid using clinical isolates from the United States (50 medical centers). Diagnostic microbiology and infectious disease. 2007;59(3):309-17.

49. Pai MP, Rodvold KA, Schreckenberger PC, Gonzales RD, Petrolatti JM, Quinn JP. Risk factors associated with the development of infection with linezolid-and vancomycin-resistant Enterococcus faecium. Clinical infectious diseases. 2002;35(10):1269-72.

50. Smith PF, Booker BM, Ogundele AB, Kelchin P. Comparative in vitro activities of daptomycin, linezolid, and quinupristin/dalfopristin against gram-positive bacterial isolates from a large cancer center. Diagnostic microbiology and infectious disease. 2005;52(3):255-9.

51. Flamm RK, Mendes RE, Hogan PA, Streit JM, Ross JE, Jones RN. Linezolid surveillance results for the United States (LEADER surveillance program 2014). Antimicrobial agents and chemotherapy. 2016;60(4):2273-80.

52. Mendes RE, Hogan PA, Streit JM, Jones RN, Flamm RK. Update on linezolid in vitro activity through the Zyvox Annual Appraisal of Potency and Spectrum Program, 2013. Antimicrobial agents and chemotherapy. 2015;59(4):2454-7.

53. Richter S, Kealey D, Murray C, Heilmann K, Coffman S, Doern G. The in vitro activity of daptomycin against Staphylococcus aureus and Enterococcus species. Journal of Antimicrobial Chemotherapy. 2003;52(1):123-7.

54. Dilworth TJ, Beck ET, Pedersen RA, Al-Karkokly WG, Cook MM, Aldag EK, et al. High rate of linezolid intermediate susceptibility and resistance among enteric vancomycin-resistant Enterococcus (VRE) recovered from hospitalized patients actively screened for VRE colonization. Infection Control & Hospital Epidemiology. 2019;40(7):821-2.

55. Li L, Dai J-x, Xu L, Chen Z-h, Li X-y, Liu M, et al. Antimicrobial resistance and pathogen distribution in hospitalized burn patients: a multicenter study in Southeast China. Medicine. 2018;97(34).

56. Bai B, Hu K, Li H, Yao W, Li D, Chen Z, et al. Effect of tedizolid on clinical Enterococcus isolates: in vitro activity, distribution of virulence factor, resistance genes and multilocus sequence typing. FEMS microbiology letters. 2018;365(3):fnx284.

57. Zhang F, Li Y, Lv Y, Zheng B, Xue F. Bacterial susceptibility in bloodstream infections: Results from China Antimicrobial Resistance Surveillance Trial (CARST) Program, 2015–2016. Journal of global antimicrobial resistance. 2019;17:276-82.

58. Chen H, Wang X, Yin Y, Li S, Zhang Y, Wang Q, et al. Molecular characteristics of oxazolidinone resistance in enterococci from a multicenter study in China. BMC microbiology. 2019;19(1):162.

59. Cai J, Schwarz S, Chi D, Wang Z, Zhang R, Wang Y. Faecal carriage of optrA-positive enterococci in asymptomatic healthy humans in Hangzhou, China. Clinical Microbiology and Infection. 2019;25(5):630. e1-. e6.

60. Zhao J, Wang Q, Zhang J. Changes in Microbial Profiles and Antibiotic Resistance Patterns in Patients with Biliary Tract Infection over a Six-Year Period. Surgical infections. 2019;20(6):480-5.

61. Zheng J-X, Wu Y, Lin Z-W, Pu Z-Y, Yao W-M, Chen Z, et al. Characteristics of and virulence factors associated with biofilm formation in clinical Enterococcus faecalis isolates in China. Frontiers in microbiology. 2017;8:2338.

62. Shi L, Wu D, Wei L, Liu S, Zhao P, Tu B, et al. Nosocomial and community-acquired spontaneous bacterial peritonitis in patients with liver cirrhosis in China: comparative microbiology and therapeutic implications. Scientific reports. 2017;7(1):1-9.

63. Huang L, Zhang R, Hu Y, Zhou H, Cao J, Lv H, et al. Epidemiology and risk factors of methicillin-resistant Staphylococcus aureus and vancomycin-resistant enterococci infections in Zhejiang China from 2015 to 2017. Antimicrobial Resistance & Infection Control. 2019;8(1):90.

64. Kang M, Xie Y, He C, Chen Z, Guo L, Yang Q, et al. Molecular characteristics of vancomycin-resistant Enterococcus faecium from a tertiary care hospital in Chengdu, China. European journal of clinical microbiology & infectious diseases. 2014;33(6):933-9.

65. Zhu Q, Yue Y, Zhu L, Cui J, Zhu M, Chen L, et al. Epidemiology and microbiology of Gram-positive bloodstream infections in a tertiary-care hospital in Beijing, China: a 6-year retrospective study. Antimicrobial Resistance & Infection Control. 2018;7(1):107.

66. Wang Q-Y, Li R-H, Shang X-H. Urinary tract infection caused by Enterococcus isolates: aetiology and antimicrobial resistance patterns. Journal of chemotherapy. 2015;27(2):117-9.

67. Hua R, Xia Y, Wu W, Yang M, Yan J. Molecular epidemiology and mechanisms of 43 low-level linezolid-resistant Enterococcus faecalis strains in Chongqing, China. Annals of laboratory medicine. 2019;39(1):36-42.

68. Su M-S, Lin M-H, Zhao Q-H, Liu Z-W, He L, Jia N. Clinical study of distribution and drug resistance of pathogens in patients with severe acute pancreatitis. Chinese medical journal. 2012;125:1772-6.

69. Qu T-T, Chen Y-G, Yu Y-S, Wei Z-Q, Zhou Z-H, Li L-J. Genotypic diversity and epidemiology of high-level gentamicin resistant Enterococcus in a Chinese hospital. Journal of Infection. 2006;52(2):124-30.

70. Zhou W, Gao S, Xu H, Zhang Z, Chen F, Shen H, et al. Distribution of the optrA gene in Enterococcus isolates at a tertiary care hospital in China. Journal of global antimicrobial resistance. 2019;17:180-6.

71. Li W, Li J, Wei Q, Hu Q, Lin X, Chen M, et al. Characterization of aminoglycoside resistance and virulence genes among Enterococcus spp. isolated from a hospital in China. International journal of environmental research and public health. 2015;12(3):3014-25.

72. Jia X, Ma W, Xu X, Yang S, Zhang L. Retrospective analysis of hospital-acquired linezolid-nonsusceptible enterococci infection in Chongqing, China, 2011-2014. American journal of infection control. 2015;43(12):e101-e6.

73. Zhang Y, Dong G, Li J, Chen L, Liu H, Bi W, et al. A high incidence and coexistence of multiresistance genes cfr and optrA among linezolid-resistant enterococci isolated from a teaching hospital in Wenzhou, China. European Journal of Clinical Microbiology & Infectious Diseases. 2018;37(8):1441-8.

74. Tian Y, Li T, Zhu Y, Wang B, Zou X, Li M. Mechanisms of linezolid resistance in staphylococci and enterococci isolated from two teaching hospitals in Shanghai, China. BMC microbiology. 2014;14(1):292.

75. Phukan C, Lahkar M, Ranotkar S, Saikia KK. Emergence of vanA gene among vancomycin-resistant enterococci in a tertiary care hospital of North-East India. The Indian journal of medical research. 2016;143(3):357.

76. Bhatt P, Patel A, Sahni A, Praharaj A, Grover N, Chaudhari C, et al. Emergence of multidrug resistant enterococci at a tertiary care centre. medical journal armed forces india. 2015;71(2):139-44.

77. Yadav G, Thakuria B, Madan M, Agwan V, Pandey A. Linezolid and vancomycin resistant enterococci: a therapeutic problem. Journal of clinical and diagnostic research: JCDR. 2017;11(8):GC07.

78. Jahansepas A, Ahangarzadeh Rezaee M, Hasani A, Sharifi Y, Rahnamaye Farzami M, Dolatyar A, et al. Molecular Epidemiology of Vancomycin–Resistant Enterococcus faecalis and Enterococcus faecium Isolated from Clinical Specimens in the Northwest of Iran. Microbial Drug Resistance. 2018;24(8):1165-73.

79. Esmailzadeh M, Moniri R. Distribution of gentamicin resistant genes of nosocomial Enterococcus spp from Intensive Care Unit of Shahid Beheshty Hospital in Kashan, Iran. An International Peer Reviewed Open Access Journal For Rapid Publication. 2016:149.

80. Doust R, Fard S, Mobarez A. Detection of linezolid-resistant, vancomycin-resistant Enterococcus strain in Iran: P1834. Clinical Microbiology & Infection. 2007;13.

81. Yasliani S, Mobarez A, Doust R, Satari M, Teymornejad O. Linezolid vancomycin resistant Enterococcus isolated from clinical samples in Tehran hospitals. Indian journal of medical sciences. 2009;63(7):297.

82. Coskun USS. Investigation of the relationship between virulence factors and antibiotic resistance of Enterococci isolates. Cell Mol Biol (Noisy le Grand). 2019;65(2).

83. Cakirlar FK, Günaydın M, Gonullu N, Kiraz N. The species distribution and resistance pattern of vancomycin resistance enterococci from bloodstream infections in Istanbul, Turkey. International Journal of Infectious Diseases. 2016;45:98.

84. Sibel A, Köroglu M, Muharrem A. The evaluation of antimicrobial susceptibility of urine enterococci with the Vitek 2 automated system in eastern Turkey. Southeast Asian Journal of Tropical Medicine & Public Health. 2012;43(4):986-91.

85. Verma N, Clarke RW, Bolton-Maggs PH, van Saene HK. Gut overgrowth of vancomycin-resistant enterococci (VRE) results in linezolid-resistant mutation in a child with severe congenital neutropenia: a case report. Journal of pediatric hematology/oncology. 2007;29(8):557-60.

86. Decousser J-W, Woerther P-L, Soussy C-J, Fines-Guyon M, Dowzicky MJ. The tigecycline evaluation and surveillance trial; assessment of the activity of tigecycline and other selected antibiotics against gram-positive and gram-negative pathogens from France collected between 2004 and 2016. Antimicrobial Resistance & Infection Control. 2018;7(1):68.

87. Sassi M, Guérin F, Zouari A, Beyrouthy R, Auzou M, Fines-Guyon M, et al. Emergence of optrA-mediated linezolid resistance in enterococci from France, 2006–16. Journal of Antimicrobial Chemotherapy. 2019;74(6):1469-72.

88. Sader HS, Streit J, Fritsche T, Jones R. Antimicrobial susceptibility of gram-positive bacteria isolated from European medical centres: results of the Daptomycin Surveillance Programme (2002–2004). Clinical microbiology and infection. 2006;12(9):844-52.

89. Schulte B, Heininger A, Autenrieth I, Wolz C. Emergence of increasing linezolid-resistance in enterococci in a post-outbreak situation with vancomycin-resistant Enterococcus faecium. Epidemiology & Infection. 2008;136(8):1131-3.

90. Bodmann K-F, Heizmann WR, Von Eiff C, Petrik C, Löschmann P-A, Eckmann C. Therapy of 1,025 severely ill patients with complicated infections in a German multicenter study: safety profile and efficacy of tigecycline in different treatment modalities. Chemotherapy. 2012;58(4):282-94.

91. Theilacker C, Jonas D, Huebner J, Bertz H, Kern W. Outcomes of invasive infection due to vancomycin-resistant Enterococcus faecium during a recent outbreak. Infection. 2009;37(6):540.

92. Werner G, Essig A, Bartel M, Wellinghausen N, Klare I, Witte W, et al. P1626 Detection of resistance to linezolid in Enterococcus spp. by fluorescence in situ hybridisation using locked nucleic acid probes. International Journal of Antimicrobial Agents. 2007(29):S457-S8.

93. Brauers J, Kresken M, Hafner D, Shah P, Group GLRS. Surveillance of linezolid resistance in Germany, 2001–2002. Clinical microbiology and infection. 2005;11(1):39-46.

94. Klare I, Fleige C, Geringer U, Thürmer A, Bender J, Mutters NT, et al. Increased frequency of linezolid resistance among clinical Enterococcus faecium isolates from German hospital patients. Journal of global antimicrobial resistance. 2015;3(2):128-31.

95. Kresken M, Leitner E, Brauers J, Geiss H, Halle E, von Eiff C, et al. Susceptibility of common aerobic pathogens to tigecycline: results of a surveillance study in Germany. European journal of clinical microbiology & infectious diseases. 2009;28(1):83-90.

96. Campanile F, Bongiorno D, Mongelli G, Zanghì G, Stefani S. Bactericidal activity of ceftobiprole combined with different antibiotics against selected Gram-positive isolates. Diagnostic microbiology and infectious disease. 2019;93(1):77-81.

97. Manfredi R, Nanetti A, Valentini R, Morelli S, Calza L. A 2-year survey of bacteriologic profile and antimicrobial susceptibility levels of enterococci in a large Italian teaching hospital. Infectious Diseases in Clinical Practice. 2004;12(3):163-70.

98. Rodloff A, Leclercq R, Debbia E, Cantón R, Oppenheim B, Dowzicky M. Comparative analysis of antimicrobial susceptibility among organisms from France, Germany, Italy, Spain and the UK as part of the tigecycline evaluation and surveillance trial. Clinical microbiology and infection. 2008;14(4):307-14.

99. Borbone S, Lupo A, Mezzatesta ML, Campanile F, Santagati M, Stefani S. Evaluation of the in vitro activity of tigecycline against multiresistant Gram-positive cocci containing tetracycline resistance determinants. International journal of antimicrobial agents. 2008;31(3):209-15.

100. Cercenado E, Marin M, Cuevas O, Bouza E. P889 Emergence of linezolid-resistant Enterococcus faecalis in Spain and rapid characterisation by real-time PCR. International Journal of Antimicrobial Agents. 2007(29):S230.

101. Johnson A, Mushtaq S, Warner M, Livermore D. Activity of daptomycin against multi-resistant Gram-positive bacteria including enterococci and Staphylococcus aureus resistant to linezolid. International journal of antimicrobial agents. 2004;24(4):315-9.

102. Bassetti M, Farrel PA, Callan DA, Topal JE, Dembry LM. Emergence of linezolid-resistant Enterococcus faecium during treatment of enterococcal infections. Int J Antimicrob Agents. 2003;21(6):593-4.

103. Sami H, Singh A, Ahmed S, Shahid M. Emergence of linezolid resistance in Enterococci: prevalent genotypes and resistance pattern in vancomycin-resistant Enterococci in a North-Indian tertiary care hospital. New Zealand Journal of Medical Laboratory Science. 2020;74(1):27.

104. Knight T, Gupte A, Dulay K, Mitchell R, Salimnia H, Wang ZJ. Evaluation of an empiric antibiotic regimen in pediatric oncology patients presenting with fever does not reveal the emergence of antibiotic resistance over a 12-year period. Pediatric Hematology Oncology Journal. 2020;5(3):80-8.

105. Zou J, Xia Y. Molecular characteristics and risk factors associated with linezolid-resistant Enterococcus faecalis infection in Southwest China. Journal of Global Antimicrobial Resistance. 2020;22:504-10.

106. Rana D, Sande S. Study of Prevalence and Antimicrobial Susceptibility Pattern of Enterococci Isolated from Clinically Relevant Samples with Special Reference to High Level Aminoglycoside Resistance (HLAR) in a Rural Tertiary Care Hospital. Journal of Evolution of Medical and Dental Sciences. 2020;9(34):2472-9.

107. Li P, Yang Y, Ding L, Xu X, Lin D. Molecular Investigations of Linezolid Resistance in Enterococci OptrA Variants from a Hospital in Shanghai. Infection and Drug Resistance. 2020;13:2711.

108. Lewis JD, Barros AJ, Sifri CD. Comparison of risk factors and outcomes of daptomycin-susceptible and -nonsusceptible vancomycin-resistant Enterococcus faecium infections in liver transplant recipients: A reply to Jorgenson et al. 2019;21(1):e13028.

109. Rolston KV, Kapadia M, Tarrand J, Coyle E, Prince RA. Spectrum of gram-positive bacteraemia and in vitro activities of daptomycin, linezolid and vancomycin against organisms isolated from cancer patients. International journal of antimicrobial agents. 2013;41(6):516-20.

110. Patel K, Kabir R, Ahmad S, Allen SL. Assessing outcomes of adult oncology patients treated with linezolid versus daptomycin for bacteremia due to vancomycin-resistant Enterococcus. Journal of Oncology Pharmacy Practice. 2016;22(2):212-8.

111. Sader HS, Farrell DJ, Flamm RK, Jones RN. Analysis of 5-year trends in daptomycin activity tested against Staphylococcus aureus and enterococci from European and US hospitals (2009–2013). Journal of global antimicrobial resistance. 2015;3(3):161-5.

112. Kelesidis T, Humphries R, Chow AL, Tsiodras S, Uslan DZ. Emergence of daptomycin-non-susceptible enterococci urinary tract isolates. Journal of medical microbiology. 2013;62(7):1103-5.

113. Kelesidis T, Humphries R, Uslan DZ, Pegues D. De novo daptomycin-nonsusceptible enterococcal infections. Emerging infectious diseases. 2012;18(4):674.

114. Udaondo Z, Jenjaroenpun P, Wongsurawat T, Meyers E, Anderson C, Lopez J, et al., editors. Two Cases of Vancomycin-Resistant Enterococcus faecium Bacteremia With Development of Daptomycin-Resistant Phenotype and its Detection Using Oxford Nanopore Sequencing. Open forum infectious diseases; 2020: Oxford University Press US.
